# Sesquiterpenoids from the Inflorescence of *Ambrosia artemisiifolia*

**DOI:** 10.3390/molecules27185915

**Published:** 2022-09-12

**Authors:** Zhi Zeng, Hong Huang, Hualiang He, Lin Qiu, Qiao Gao, Youzhi Li, Wenbing Ding

**Affiliations:** 1 College of Plant Protection, Hunan Agricultural University, Changsha 410128, China; 2 Institute of Plant Protection, Hunan Province, Changsha 410125, China; 3 Hunan Provincial Engineering & Technology Research Center for Biopesticide and Formulation Processing, Changsha 410128, China

**Keywords:** *Ambrosia artemisiifolia*, sesquiterpenoids, allelopathy activity

## Abstract

The successful invasion of *Ambrosia artemisiifolia* is largely due to allelopathy. As an invasive alien plant, *A. artemisiifolia* has spread rapidly in Asia and Europe. Studies have shown that sesquiterpenoids play an important role in plant allelopathy. However, it is unclear whether the inflorescence of *A. artemisiifolia* also contains allelopathic components. In this paper, our phytochemical research focuses on the inflorescence of *A. artemisiifolia*. Twenty sesquiterpenoids, including four new ones (**1**–**4**) were isolated through successive chromatographic columns and identified by spectroscopic methods. At a concentration of 200 μg/mL, all the compounds tested were evaluated for their allelopathic activities on seedling growth of wheat. Our results indicate that nine compounds inhibited both the root and shoot growth of seedlings. Compounds **14**, **15**, **17,** and **20** significantly inhibited root length, which was more than 50% shorter than the control. This study identified the chemical profile of the sesquiterpenoids occurring in the inflorescence of *A. artemisiifolia*. The bioactivity screening results provide further understanding of the chemical basis of allelopathy in *A. artemisiifolia*.

## 1. Introduction

*Ambrosia artemisiifolia* L. (Asteraceae), which is native to North America, is an invasive weed widely distributed throughout temperate regions of the world [1]. This invasive plant has the potential to destroy native ecosystems and reduce agricultural yields [2]. Allelopathy describes the chemicals exuded from roots, rhizomes, leaves, stems, and/or litter of an individual and the resultant beneficial or deleterious effects on other organisms and is an important factor in the successful invasion of alien plants [3]. Meanwhile, because allelopathic components generally have strong phytotoxic activities against many weeds, the allelochemicals have the potential to develop new herbicides, which have the advantages of safety, easy degradation, and no resistance in weed control [4]. Previous studies reported that different solvent extracts of *A. artemisiifolia* had significant inhibitory effects on crop growth and seed germination, weeds, and other plants [5,6]. Studies have shown that sesquiterpenoids are the main chemical components of *Ambrosia* and play an important role in plant allelopathy [7,8,9,10]. Every year between July and August, clusters of columnar inflorescence from *A. artemisiifolia* produce large amounts of pollen causing a series of allergic reactions and affecting human health [11]. However, it is unclear whether the inflorescence of *A. artemisiifolia* also contains allelopathic components. Therefore, our phytochemical research focuses primarily on the inflorescence of *A. artemisiifolia*, from which we obtained twenty sesquiterpenoids, including four new ones (**1**–**4**). Moreover, all the compounds were evaluated for their allelopathic activities on the seedling growth of wheat.

## 2. Results and Discussions

**Figure 1:**
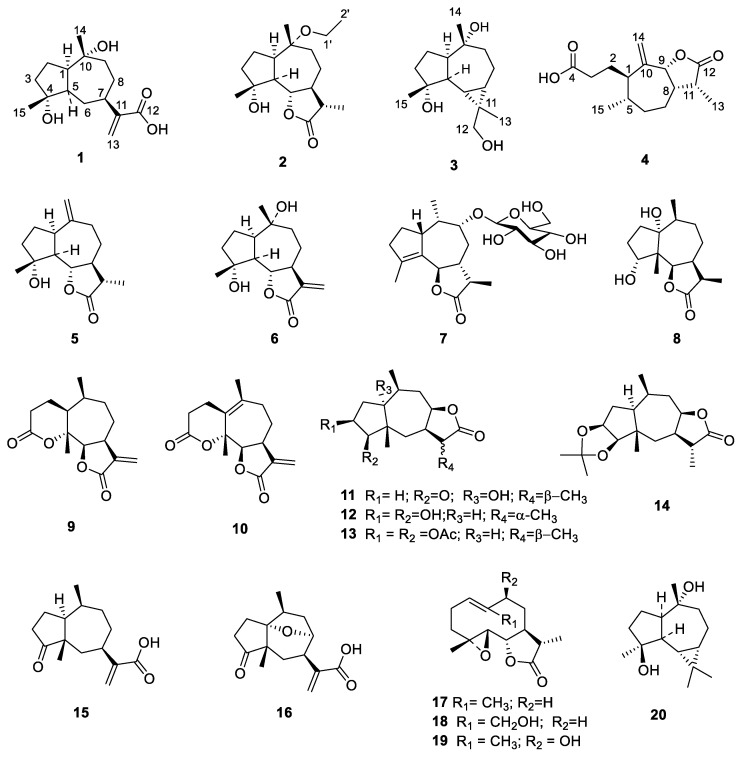
Structures of compounds **1**–**20** isolated from *A. artemisiifolia*.

Air-dried and powdered inflorescence of *A. artemisiifolia* was extracted with petroleum ether and then further extracted with ethanol. A total of 20 sesquiterpenoids (Figure 1), including four new ones (**1**–**4**), were isolated from the petroleum ether extract and ethanol extracts using chromatographic methods.

### 2.1. Chemical Characterization of the Compounds

**Figure 2:**
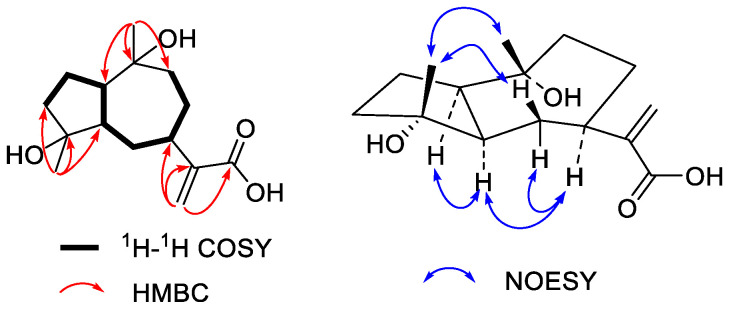
Key ^1^H-^1^H COSY, HMBC, and NOESY correlations of **1**.

Compound **1** was obtained as a yellowish syrup. The molecular formula was C_15_H_24_O_4_ as determined by the HRESIMS peak at *m/z* 267.1598 ([M − H]^−^, calcd. for 267.1596), with four degrees of unsaturation. The ^1^H NMR data of **1** (Table 1) show two singlet methyls at δ_H_ 1.50 (H_3_-14) and 1.44 (H_3_-15) and a terminal alkyne at δ_H_ 6.48 and 5.68 (H_2_-13). The ^13^C NMR (Table 2) indicated 15 resonances ascribed to two methyls, a terminal alkyne, five methylene, three methines, two oxygenated tertiary carbons, and one *α*,*β*-unsaturated carboxylic carbon. The ^1^H-^1^H COSY correlations enabled the establishment of a long coupling carbon chain from C-3 to C-1 that extended via C-5 sequentially to C-9 as shown in bold bonds (Figure 2). The HMBC correlations from H_3_-15 (δ_H_ 1.44) to C-3 (δ_C_ 40.8), C-4 (80.8), and C-5 (53.8) and from H_3_-14 (δ_H_ 1.50) to C-1 (δ_C_ 53.1), C-9 (41.7), and C-10 (73.9) established **1** as a 4,10-dihydroxylguaiane-type sesquiterpene (Figure 2). While HMBC correlations from H_2_-13 (δ_H_ 6.48 and 5.68) to C-11 (δ_C_ 150.3), C-12 (170.1), and C-7 (41.6) indicated that a conjugated acrylic acid moiety was attached at C-7. Accordingly, the planar structure of compound **1** was established as 4,10-dihydroxyguaia-11(13)-en-12-acid. The strong NOESY correlation between H-1 and H-5 indicated *cis*-fused framework between the five-carbon ring and the seven-carbon ring. NOEs, H-5 with H-7 show that all of those protons (H-1, H-5, and H-7) were cofacial and were assigned to be *α*-oriented [12]. In addition, NOEs, H_3_-14 with H_3_-15 and H_3_-15 with H*β*-6 were observed in the *cis*-fused framework suggesting CH_3_-14 and CH_3_-15 were *β*-oriented (Figure 2). Thus compound **1** was determined to be 4*β*,10*β*-dihydroxy-1*α*,5*α,*7*α*H-guaia-11(13)-en-12-acid.

**Figure 3:**
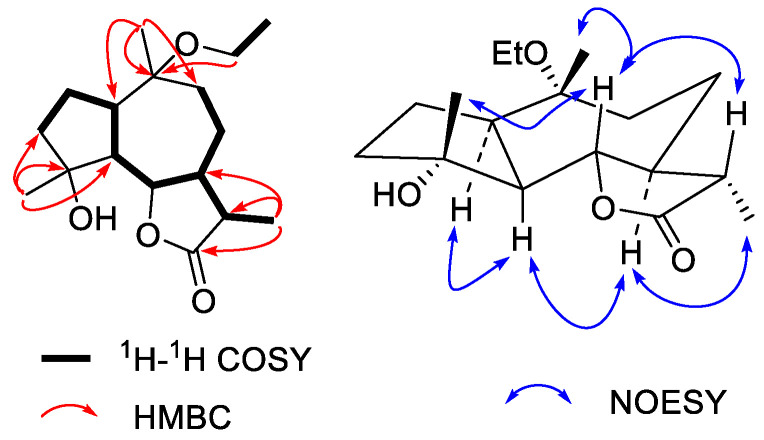
Key ^1^H-^1^H COSY, HMBC, and NOESY correlations of **2**.

Compound **2** was obtained as a colorless syrup. The molecular formula of **2** was C_17_H_28_O_4_ as determined by the HRESIMS peak at *m/z* 615.3872 ([2M +Na]^+^, calcd. for 615.3873) with four degrees of unsaturation. The ^1^H NMR data (Table 1) show signals of four tertiary methyls at δ_H_ 1.22 (d, H_3_-13), 1.15 (s, H_3_-14), 1.33 (s, H_3_-15), and 1.11 (t, H_3_-2’) and an oxygenated methine at δ_H_ 4.23 (H-6) and an oxygenated methylene at 3.37 (H_2_-1’). The ^13^C NMR data of **2** (Table 2) identify 17 resonances ascribed to four methyls, five methylenes (one oxygenated), five methines (one oxygenated), two oxygenated tertiary carbons, and one ester carbonyl carbon. The ^1^H and ^13^C NMR data of **2** (Table 1 and Table 2) display characteristic signals of a guaiane-type sesquiterpenoid, which quite resembled that of *α*-Hydroxy-10*α*-methoxyguaian-12,6-olide [13]. A careful comparison of ^1^H and ^13^C NMR data and molecular formula showed that ethoxyl, rather than methoxyl, existed at C-10 in **2**. The relative configuration of **2** was deduced from the NOE correlations (Figure 3). The cross peaks of H-1/H-5, H-5/H-7, and H-7/H_3_-13 indicated that these protons were cofacial and *α*-oriented. The cross peaks of H-6/H_3_-16, H-6/H_3_-14, and H-6/H-11 proved that they were on the opposite face and were assigned to be *β*-oriented. Accordingly, compound **2** was determined structurally as 4*α*-Hydroxy-10*α*-ethoxyguaian-12,6-olide.

**Figure 4:**
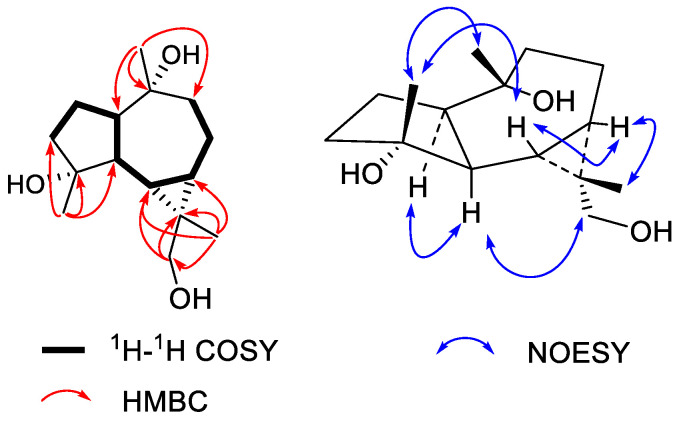
Key ^1^H-^1^H COSY, HMBC, and NOESY correlations of **3**.

Compound **3** was obtained as a colorless crystal plate The molecular formula of **3** was C_15_H_26_O_3_ as determined by the HRESIMS peak at *m/z* 253.1803 ([M − H]^−^, calcd. for 253.1804). The ^1^H NMR data (Table 1) show signals of three tertiary methyls at δ_H_ 1.42 (H_3_-13), 1.43 (H_3_-14), and 1.73 (H_3_-15). The ^13^C NMR data of **3** (Table 2) identify 15 resonances ascribed to three terminal methyls, five methylenes (one oxygenated), four methines, and three tertiary carbon (two oxygenated). Taking into account the molecular formula, a tricyclic skeleton was indicated. The presence of one characteristic quaternary carbon at high field (C-11, δ_C_ 24.9) displayed it is an aromadendrane-type sesquiterpene [14,15]. The HMBC correlations from H_2_-12 (δ_H_ 4.06 and 4.15) to C-11, C-13, C-6, and C-7 and from H_3_-13 (δ_H_ 1.42) to C-11, C-12, C-6, and C-7 verified the existence of a cyclopropyl unit at C-6 and C-7. Moreover, The HMBC correlations from H_3_-14 (δ_H_ 1.43) to C-1, C-9, and C-10 and from H_3_-15 (δ_H_ 1.73) to C-3, C-4, and C-5 established the planar structure of compound 165 as aromadendrane-4,10,12-triol. The relative configurations of the six chiral centers at C-1, C-4, C-5, C-6, C-7, and C-9 in **3** were similar to those in (−)-alloaromadendrane-4*β*,10*β*-diol [16], which was confirmed by the ROESY correlations of H-1with H-5 and H_2_-12; H-6 with H_3_-11; and H-7 with H-8 in its ROESY spectrum (Figure 4). Therefore, compound **3** was characterized as (−)-alloaromadendrane-4*β*,10*β,12*-triol.

**Figure 5:**
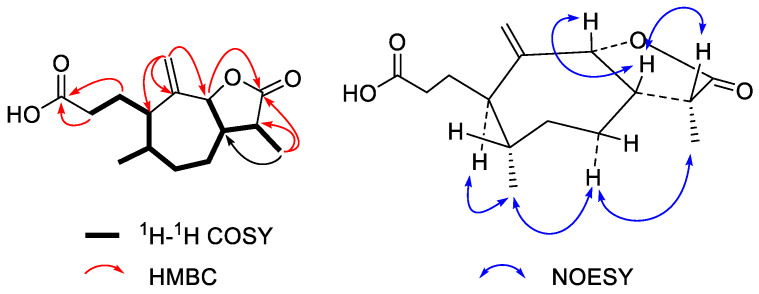
Key ^1^H-^1^H COSY, HMBC, and NOESY correlations of **4**.

Compound **4** was obtained as a colorless viscous oil. The molecular formula of **4** was C_15_H_22_O_4_ as determined by the HRESIMS peak at *m/z* 265.1441 ([M − H]^+^, calcd. for 265.1440). In ^1^H NMR, it can be recognized that there are two methyl groups at δ_H_ 1.11 and 0.75 (each d, 3H) and an exocyclic methylene at δ_H_ 5.64 and 4.93. The ^13^C NMR spectrum indicated 15 carbon resonances (Table 2), which contained two carbonyl carbons, two downfield alkene carbons, an oxygenated carbon, two methyls, two methylenes, and five methines. A long coupling carbon chain from C-3 to C-9 that extended from C-8 to C-13 was deduced by the ^1^H–^1^H COSY correlations (Figure 5). The HMBC correlations from H_3_-13 to C-8, C-11, and C-12 and from H-9 to C-12, as well as from H_3_-14 to C-1, C-9, and C-10, established a preguaiane-type sesquiterpene containing α-methyl-γ-lactone. The relative configuration of **4** was concluded from the NOESY experiment (Figure 5). The cross peaks of H-1/H_3_-15, H-7α/ H_3_-15, and H-7α/ H_3_-13 indicated that they were cofacial and *α*-oriented. The cross peaks of H-8/H-9 and H-8/H-11 sustained that these protons were on the other face and assigned to be *β*-oriented. Therefore, compound **4** was characterized as depicted and named preambrosia A.

Known compounds were identified by comparison with the experimental and reported spectroscopic data as follows: (-)-compressanolide (**5**) [17], magnograndiolide (**6**) [18], 1*β*,7*β*,9*β*,10*β*,13*α*H-guaia-4(5)-en-12,6*β*-olide-9-*O*-*β*-d-glucoside (**7**) [19], tetrahydrocoropilin (**8**) [8], psilostachyin C (**9**) [20], psilostachyin B (**10)** [21], 11*α*,13-dihydroperuvin (**11**) [22], 3*α*,4*β*-dihydrocumanin (**12**) [23], dihydrocumanin diacetate (**13**) [23], dihydrocumanin acetonide (**14**) [23], damsinic acid (**15**) [24], ambrosic acid (**16**) [25], 11,13-dihydroparthenolide (**17**) [26], 11*β*,13-dihydro-14-hydroxyparthenolide (**18**) [26], (-)-9α-hydroxydihydroparthenolid (**19**) [26], and (+)-alloaromadendrane-4*β*,10*α*-diol (**20**) [27]. The stucture of these isolated sesquiterpeniods can be divided into guaiane-, pseudoguaiane-, secopseudoguaiane-, germacrene-, and aromadendrane-type carbon skeletons. Among them, guaiane- and pseudoguaiane-type sesquiterpeniods are the most characteristic and diverse subclass in the inflorescence of *A. artemisiifolia*. Aromadendrane-type sesquiterpenes are rare in the genus *Ambrosia* [28]; however, aromadendraniols **3** and **20** were isolated from the inflorescence of *A. artemisiifolia.*

### 2.2. Allelopathy Activity

**Figure 6:**
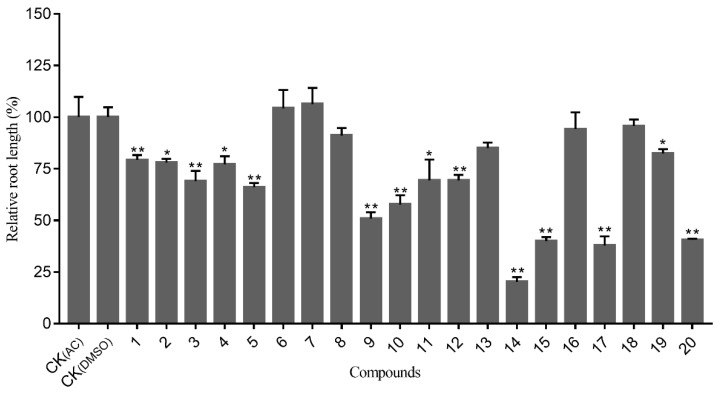
Allelopathic activity of sesquiterpenoids (**1**–**20**) on root length of wheat. Note: CK (_AC_) = acetone solvent control and CK (_DMSO_) = DMSO solvent control; the compounds were solubilized with acetone, except **1**, **7**, and **9** were solubilized with DMSO. Each compound’s result was compared with the solvent control via independent sample *t*-test; * indicates significant difference (*P* < 0.05); ** indicates extremely significant difference (*P* < 0.01).

**Figure 7:**
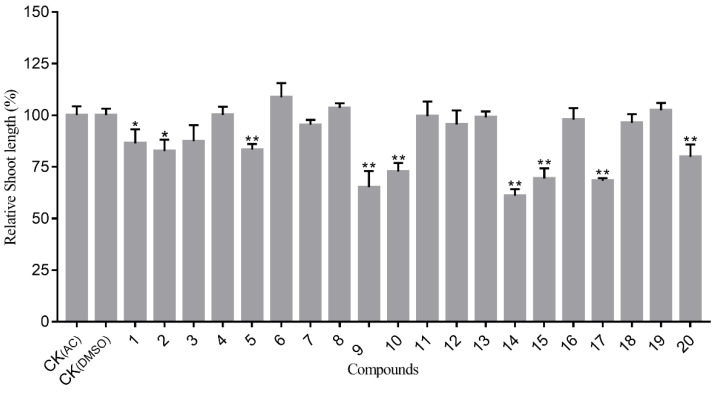
Allelopathic activity of sesquiterpenoids (**1**–**20**) on shoot length of wheat. Note: CK (_AC_) = acetone solvent control and CK (_DMSO_) = DMSO solvent control; the compounds were solubilized with acetone, except **1**, **7**, and **9** were solubilized with DMSO. Each compound’s result was compared with the solvent control via independent sample *t*-test; * indicates significant difference (*P* < 0.05); ** indicates extremely significant difference (*P* < 0.01).

Compounds **1**–**20** were evaluated for their allelopathic activities on the seedling growth of wheat (*T. Aestivum*) at a concentration of 200 μg/mL. Our results show that fourteen compounds (**1**–**4**, **5**, **9**–**12**, **14**–**15**, **17**, and **19**–**20**) inhibited the root growth of the tested seedlings to varying degrees. Compounds **14**, **15**, **17,** and **20** significantly inhibited root length, which was more than 50% shorter than that of the control (Figure 6). In contrast, the inhibitory effect of these active compounds on shoot growth was reasonably weak, with relative shoot length being no less than 60% of the control (Figure 7). It is notable that compound **14**, a pseudoguaiane-type sesquiterpene, showed the most potent allelopathic activity both on root and shoot length. Comparing the structure–activity relationships of compounds **11**–**14** showed that **14** had an acetonide group on the adjacent hydroxyl of C-3/C-4, which may be related to a high allelopathic effect. Previous research has demonstrated that parthenolides, germacrene-type sesquiterpenes, have strong allelopathic inhibitory activity [29]. The structural analogue of **17** also showed good inhibitory activity, but compounds **18** and **19** with an additional hydroxyl at C-14 and C-9, respectively, exhibited very weak allelopathic activities.

Sesquiterpenoids, which play an important role in plant allelopathy, are abundant in many invasive plants and can significantly inhibit the growth of neighboring plants [9,10]. On the other hand, allelopathic components generally have strong phytotoxic activities against many weeds [30,31]. Our findings reveal the chemical profile of the sesquiterpenoids occurring in the inflorescence of *A. artemisiifolia*, and our bioassay results are useful in understanding the chemical basis of allelopathy in *A. artemisiifolia*.

## 3. Materials and Methods

### 3.1. General

Melting points were obtained on a SGW X-4 micromelting point apparatus (INESA Co., Shanghai, China). Optical rotations were measured on SGW-533 automatic polarimeter (INESA Co., Shanghai, China). HRESIMS spectra were taken on an API QSTAR mass spectrometer (Applied Biosystem/MSD Sciex, Concord, ON, Canada). The 1D- and 2D-NMR spectra were recorded on a Bruker Avance III 600MHz NMR spectrometer using TMS as an internal standard. Column chromatography was performed on silica gel (100–200 mesh, Qingdao Marine Chemical Ltd., Qingdao, China). Preparative TLC plates (HSGF254, Jiangyou silicone Development Co., Ltd., Yantai, China), Sephadex LH-20 (GE Healthcare, Uppsala, Sweden), and Develosil ODS (50 μm, Nomura Chemical Co. Ltd., Osaka, Japan) were used for the isolation experiments. Preparative HPLC was performed on a Waters 1525 Binary HPLC pump and a Waters 2414 refractive index detector (Waters Corp, Milford, MA, USA) using a YMC-Pack ODS-A column (250 mm ×10 mm I.D.; S-5 μm, 12 nm).

### 3.2. Plant Material

The inflorescence of *A. artemisiifolia* was collected from Cangwu County, Guangxi province, China, in August 2019, which was identified by Prof. Dai-gui Zhang (Key laboratory of Plant Resources Conservation and Utilization, Jishou University). A voucher specimen (zdg20190801) was deposited in Hunan Agricultural University. Seeds of *Triticum aestivum* L. were purchased from a seed company (Jiangsu Dingsheng Seed Co., Jiangsu, China).

### 3.3. Extraction and Isolation

The air-dried and powdered inflorescence of *A. artemisiifolia* (9.0 kg) was extracted with petroleum ether (10 L × 4) and then extracted with ethanol (95%, 10 L × 4)). The ethanol extract was suspended in water and partitioned with EtOAc and *n*-butanol successively, as described in our previous research [32].

The petroleum ether extract (254.9 g) was subjected to silica gel column chromatography (CC, 100–200 mesh, 2000 g) eluted successively with gradient petroleum ether–acetone mixtures of increasing polarity (100:0 → 60:40, v/v) to separate fifteen fractions (PE.A1–PE.A15). A large number of crystals precipitated in PE.A8, and compound **17** (1100.4 mg) was obtained by recrystallization.

The EtOAc extract (370.0 g) was separated into twelve fractions (ET.A1–ET.A12) by a silica gel column (100–200 mesh, 1000 g) eluted with an increasing gradient of CHCl_3_–MeOH mixtures.

Fraction ET.A2 (165.7 g) was decolorized on an MCI gel column to obtain a yellowish syrup subfraction A2B (133.0 g) from the 90% MeOH eluent. A2B was then subjected to silica gel CC (200–300 mesh, 1000 g) eluted carefully with a gradient of petroleum ether–acetone mixtures, yielding nine fractions Fr. B1–B9. These fractions (B1–B9) were first chromatographed on an ODS-C18 column eluting with MeOH-H_2_O (30:70→90:10, v/v) to produce seven subfractions. Each of the subfractions was further purified by a sephadex LH-20 column eluting with MeOH to collect the points with color features of terpenoids on TLC. Finally, three major points were obtained from subfraction B2 (10.3 g), of which compound **15** (157.4 mg) was directly crystallized after sephadex LH-20 CC, and compounds **5** (15.3 mg, Rt = 17 min) and **3** (103.2 mg, Rt = 28 min) were yielded by semipreparative HPLC (55% MeOH /H_2_O, 3 mL/min). Two major points were obtained from subfraction B3 (12.8 g), yielding compounds **13** (73.1 mg, R*f* = 0.3) and **2** (12.6 mg, R*f* = 0.5) by TLC preparation with CHCl_3_-MeOH (90:10) as the developing solvent. Four major points were obtained from subfraction B4 (20.4 g), and compounds **16** (571.8 mg) and **9** (43.6 mg) were crystallized after sephadex LH-20 CC; compounds **8** (22.5 mg, R*f* = 0.4) and **11** (10.2 mg, R*f* = 0.6) were yielded by TLC preparation using CHCl_3_-MeOH (95:5) as the developing solvent. Two major points were obtained from subfraction B5 (12.8 g), which obtained compounds **4** (24.8 mg, R*f* = 0.2) and **20** (12.2 mg, R*f* = 0.5) by TLC preparation with petroleum ether–acetone (90:10) as the developing solvent. Three major points were obtained from subfraction B6 (11.8 g), and compound **12** (238 mg) was crystallized after sephadex LH-20 CC; compounds **1** (15.8 mg, Rt = 36 min) and **14** (20.0 mg, Rt = 45 min) were yielded by semipreparative HPLC (45% MeOH /H_2_O, 3 mL/min). Three major points were obtained from subfraction B7 (10.5 g), and compounds **6** (13.0 mg, Rt = 18 min), **18** (15.1 mg, Rt = 22 min), and **19** (28.2 mg, Rt = 30 min) were yielded by semipreparative HPLC (30% MeOH/H_2_O, 3 mL/min).

Similarly, fractions ET.A9 (6.3 g) and ET.A10 (6.8 g) were chromatographed on ODS-C18 followed by Sephadex LH-20 (MeOH) to collect clear spots using TLC detection. Compound **10** (5.8 mg) was obtained from ET.A9 by TLC preparation with ether–acetone (90:15) as the developing solvent, while compound **7** (15.3 mg) was obtained from ET.A10 by TLC preparation using ether–acetone (80:20) as the developing solvent.

### 3.4. Physical and Chemical Data of ***1**–**4***

Compound **1:** yellowish syrup, [*α*]${}_{D}^{25}$ − 62.0 (*c* 0.6, MeOH); ^1^H NMR (600 MHz, C_5_D_5_N) and ^13^C NMR (150 MHz, C_5_D_5_N) spectroscopic data, see Table 1 and Table 2; positive ion ESIMS *m/z*: 291 [M + Na]^+^; negative ESIMS *m/z*: 267 [M − H]^−^; HRESIMS *m/z*: 267.1598 [M − H]^−^ (calcd. for C_15_H_23_O_4_, 267.1596). (Data from Figures S1–S7).

Compound **2:** colorless syrup, [*α*]${}_{D}^{25}$ − 43.0 (*c* 0.8, MeOH); ^1^H NMR (600 MHz, CDCl_3_) and ^13^C NMR (150 MHz, CDCl_3_) spectroscopic data, see Table 1 and Table 2; positive ion ESIMS *m/z*: 319 [M + Na]^+^, 615 [2M + Na]^+^; HRESIMS *m/z*: 615.3872 [2M + Na]^+^ (calcd. for C_34_H_56_O_8_Na, 615.3873). (Data from Figures S8–S14).

Compound **3**: colorless crystal plate, mp 305–307 °C, [*α*]${}_{D}^{25}$ + 14.0 (*c* 0.1, MeOH); ^1^H NMR (600 MHz, C_5_D_5_N) and ^13^C NMR (150 MHz, C_5_D_5_N) spectroscopic data, see Table 1 and Table 2; negative ESIMS *m/z*: 253 [M − H]^−^, 289 [M + Cl]^−^; HRESIMS *m/z*: 253.1803 ([M − H]^−^ (calcd. for C_15_H_25_O_3_, 253.1804). (Data from Figures S15–S21).

Compound **4:** colorless viscous oil, [*α*]${}_{D}^{25}$ − 26.0 (*c* 0.6, MeOH); ^1^H NMR (600 MHz, C_5_D_5_N) and ^13^C NMR (150 MHz, C_5_D_5_N) spectroscopic data, see Table 1 and Table 2; positive ion ESIMS *m/z*: 267 [M + H]^+^, 289 [M + Na]^+^; negative ESIMS *m/z*: 265 [M − H]^−^, 301 [M + Cl]^−^; HRESIMS *m/z*: 265.1441 ([M − H]^−^ (calcd. for C_15_H_21_O_4_, 265.1440). (Data from Figures S22–S28).

### 3.5. Bioassays

Inhibitory activities on the growth of *T. aestivum* seedlings were evaluated using the plate culture method [33]. To enhance the germination rate, *T. aestivum* seeds were submerged in a 0.3% potassium permanganate solution for 15 min before being thoroughly rinsed with distilled water. After skin breaking, *T. aestivum* seeds were germinated on filter paper in the dark at 25 °C for 24 h. After being dissolved in acetone or DMSO, each compound (**1**–**20**) was prepared as a stock solution of 2 mg/mL. The stock solutions were diluted with distilled water (containing 1% Tween 80) to obtain concentrations of 200 μg/mL. The same volume of acetone or DMSO was added to distilled water (containing 1% Tween 80) as a control. Following germination, uniformly growing seedlings (10 seeds) were transferred to 9 cm diameter Petri dish lined with filter paper. Each dish was treated with 7 mL of the prepared corresponding concentration of test solution (or control solution). Each treatment was replicated 3 times. The seedlings were then incubated in a constant temperature humidity chamber in the dark at 25 °C for 72 h. At the end of the experiment, root and stem lengths were measured.

### 3.6. Statistical Analysis

All data were subjected to analysis of variance by use of SPSS 18.0. Significant differences in seedling growth between treatment and control were calculated by one-way analysis of variance (ANOVA). Relative length (percent) was determined by the formula [treated length)/control length] × 100.

## Figures and Tables

**Table 1:** ^1^H NMR spectrum (600 MHz) data of compounds **1**–**4**.

NO.	1	2	3	4
	δ_H,_ mult (*J* in Hz)	δ_H,_ mult (*J* in Hz)	δ_H,_ mult (*J* in Hz)	δ_H,_ mult (*J* in Hz)
1	3.14, td (10.3, 8.6)	2.79, ddd (12.1, 10.7, 8.1)	1.90, m	2.21, m
			1.79, m	/
2	2.16, m	1.84, m	2.05, m	2.12, dddd (14.0, 10.1, 8.6, 5.5)
	1.82, m	1.55, m		1.90, m
3	2.05, dt (12.0, 8.8)	1.76, m	/	2.58, ddd (15.8, 8.8, 5.5)
	1.90, ddd (11.8, 8.5, 2.7)	1.77, m		2.49, ddd (15.7, 8.6, 7.0)
4	/	/	2.56, dd (9.5, 4.4)	/
5	2.69, ddd (13.4, 9.9, 3.6)	2.23, t (12.1)	0.44, t (9.5)	1.88, m
6	2.19, m	4.23, dd (11.6, 10.1)	0.91, ddd (12.1, 9.5, 5.6)	1.52, m
	1.79, m			1.36, m
7	3.33, td (10.8, 3.9)	1.81, m	2.26, m	1.15, m
	/		1.93, m	1.01, td (13.3, 11.5)
8	2.22, m	1.96, m	1.89, m	2.40, m
	1.80, m	1.32, m		
9	2.26, m	1.94, m	/	4.92, m
	1.96, ddd (12.6, 9.5, 3.7)	1.63, td (14.0, 13.4, 4.6)		
10	/	/	3.36, ddd (12.3, 7.2, 4.5)	/
11	/	2.20, m	/	2.90, m
12	/	/	4.15, d (11.1)	/
			4.06, d (11.1)	
13	6.48, d (1.6)	1.22, d (7.0)	1.42, s	1.11, d (7.4)
	5.68, d (1.6)			
14	1.50, s	1.15, s	1.43, s	5.64, d (1.5)
				4.93 d, (1.5)
15	1.44, s	1.33, s	1.73, s	0.75, d (7.0)
1′		3.37, m		
2′		1.11, t (7.0)		

Compounds **1**, **3,** and **4** were measured in C_5_D_5_N, and **2** was measured in CDCl_3_.

**Table 2:** ^13^C NMR spectrum (150 MHz) data of compounds **1**–**4**.

NO.	1	2	3	4
	δc, DEPT	δc, DEPT	δc, DEPT	δc, DEPT
1	53.1 CH	46.5 CH	25.7 CH_2_	43.9 CH
2	26.5 CH_2_	25.6 CH_2_	38.4 CH_2_	29.2 CH_2_
3	40.8 CH_2_	39.3 CH_2_	81.2 C	33.0 CH_2_
4	80.8 C	80.3 C	47.8 CH	176.2 C
5	53.8 CH	55.1 CH	26.7 CH	34.7 CH
6	33.2 CH_2_	83.0 CH	29.4 CH	37.4 CH_2_
7	41.6 CH	51.1 CH	19.5 CH_2_	18.5 CH_2_
8	31.7 CH_2_	25.9 CH_2_	39.2 CH_2_	44.8 CH
9	41.7 CH_2_	37.9 CH_2_	73.2 C	83.1 CH
10	73.9 C	78.0 C	54.5 CH	143.9 C
11	150.3 C	41.4 CH	24.9 C	39.7 CH
12	170.1 C	178.0 C	63.0 CH_2_	178.6 C
13	121.4 CH_2_	12.9 CH_3_	24.7 CH_3_	10.9 CH_3_
14	28.0 CH_3_	22.5 CH_3_	32.6 CH_3_	111.5 CH_2_
15	24.9 CH_3_	24.0 CH_3_	26.4 CH_3_	14.0 CH_3_
1′		55.4 CH_2_		
2′		16.2 CH_3_		

Compounds **1**, **3,** and **4** were measured in C_5_D_5_N, and **2** was measured in CDCl_3_.

## Data Availability

All data included in this study are available upon request by contact with the corresponding author.

## Supplementary Materials

The following supporting information can be downloaded at: https://www.mdpi.com/article/10.3390/molecules27185915/s1, Figures S1–S7: HR-ESIMS, 1D and 2D-NMR spectra of compound **1**; Figures S8–S14: HR-ESIMS, 1D and 2D-NMR spectra of compound **2**; Figures S15–S21: HR-ESIMS, 1D and 2D-NMR spectra of compound **3**; Figures S22–S28: HR-ESIMS, 1D and 2D-NMR spectra of compound **4**.

## Sample Availability

Samples of the compounds are not available from the authors.
