# Supplementary material for: Sesquiterpenoids from the Inflorescence of Ambrosia artemisiifolia
Source: Molecules. 2022 Sep 12;27(18):5915. doi: 10.3390/molecules27185915 (PMC9503122; doi:10.3390/molecules27185915)
Supplement: Supplementary file 1 [file molecules-27-05915-s001.zip › molecules-1881854-Supplementary.pdf]

Supporting Information

Sesquiterpenoids from the Inflorescence of *Ambrosia artemisiifolia*

By: Zhi Zeng, Hong Huang, Hua-liang He, Lin Qiu, Qiao Gao, You-zhi Li and Wen-bing Ding

Figure List:

**Figure S1.** HR-ESIMS of compound **1**	**Figure S15.** HR-ESIMS of compound **3**
**Figure S2.** ^{1}H-NMR of compound **1**	**Figure S16.** ^{1}H-NMR of compound **3**
**Figure S3.** ^{13}C-NMR of compound **1**	**Figure S17.** ^{13}C-NMR of compound **3**
**Figure S4.** ^{1}H-^{1}H COSY of compound **1**	**Figure S18.** ^{1}H-^{1}H COSY of compound **3**
**Figure S5.** HSQC of compound **1**	**Figure S19.** HSQC of compound **3**
**Figure S6.** HMBC of compound **1**	**Figure S20.** HMBC of compound **3**
**Figure S7.** NOESY of compound **1**	**Figure S21.** NOESY of compound **3**
**Figure S8.** HR-ESIMS of compound **2**	**Figure S22.** HR-ESIMS of compound **4**
**Figure S9.** ^{1}H-NMR of compound **2**	**Figure S23.** ^{1}H-NMR of compound **4**
**Figure S10.** ^{13}C-NMR of compound **2**	**Figure S24.** ^{13}C-NMR of compound **4**
**Figure S11.** ^{1}H-^{1}H COSY of compound **2**	**Figure S25.** ^{1}H-^{1}H COSY of compound **4**
**Figure S12.** HSQC of compound **2**	**Figure S26.** HSQC of compound **4**
**Figure S13.** HMBC of compound **2**	**Figure S27.** HMBC of compound **4**
**Figure S14.** NOESY of compound **2**	**Figure S28.** NOESY of compound **4**

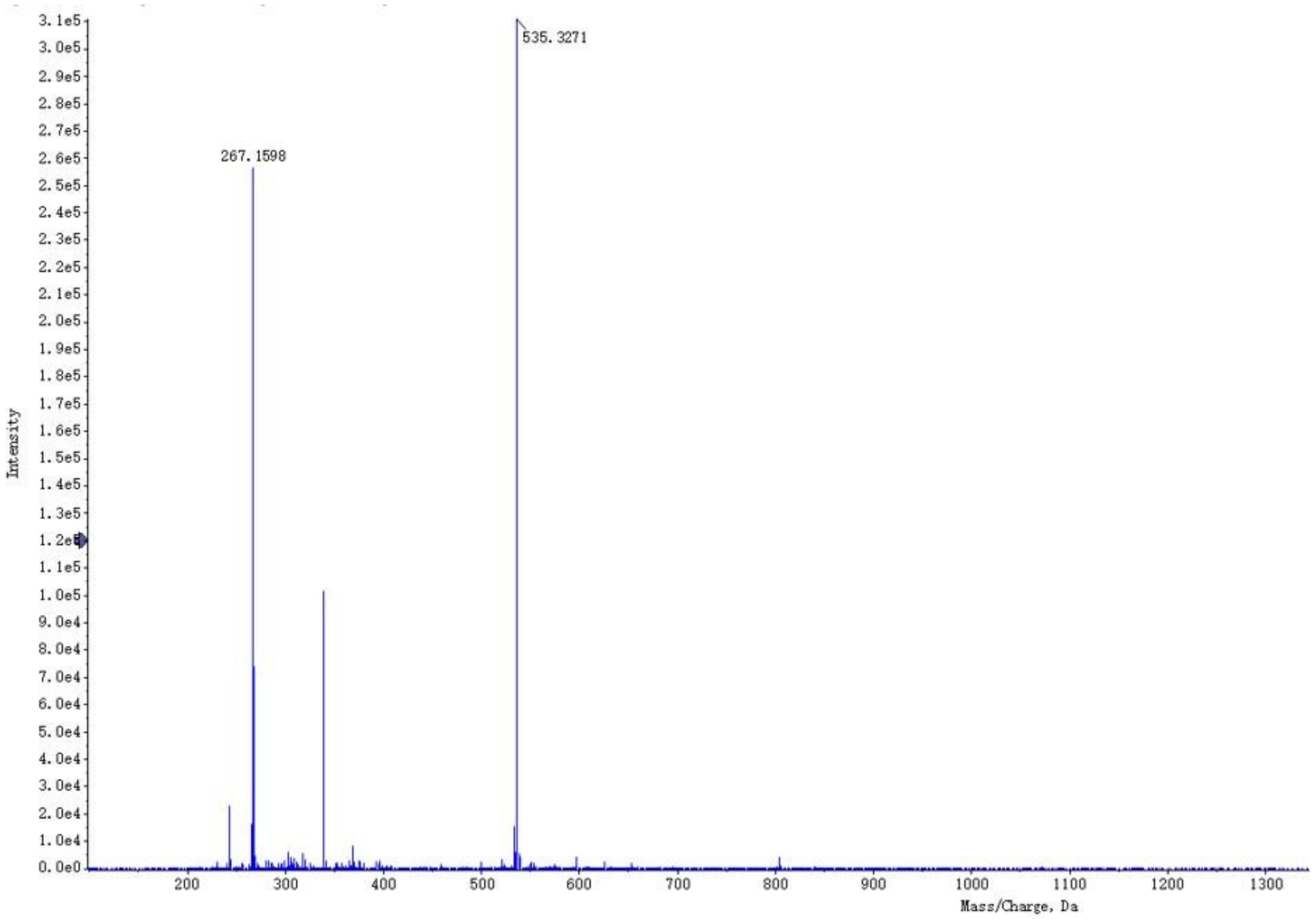

Figure S1. HR-ESIMS of compound **1**

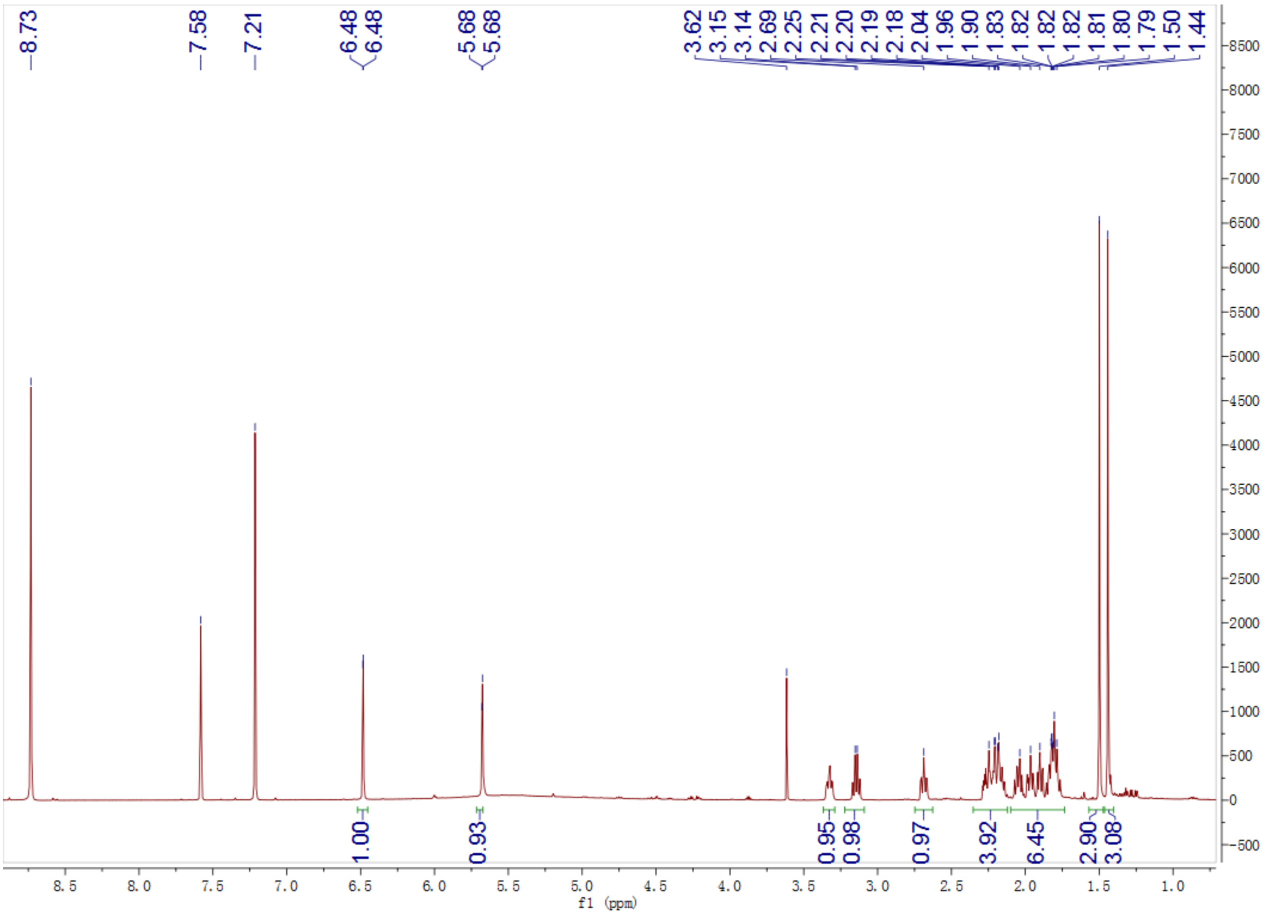

Figure S2. ^{1}H-NMR of compound **1**

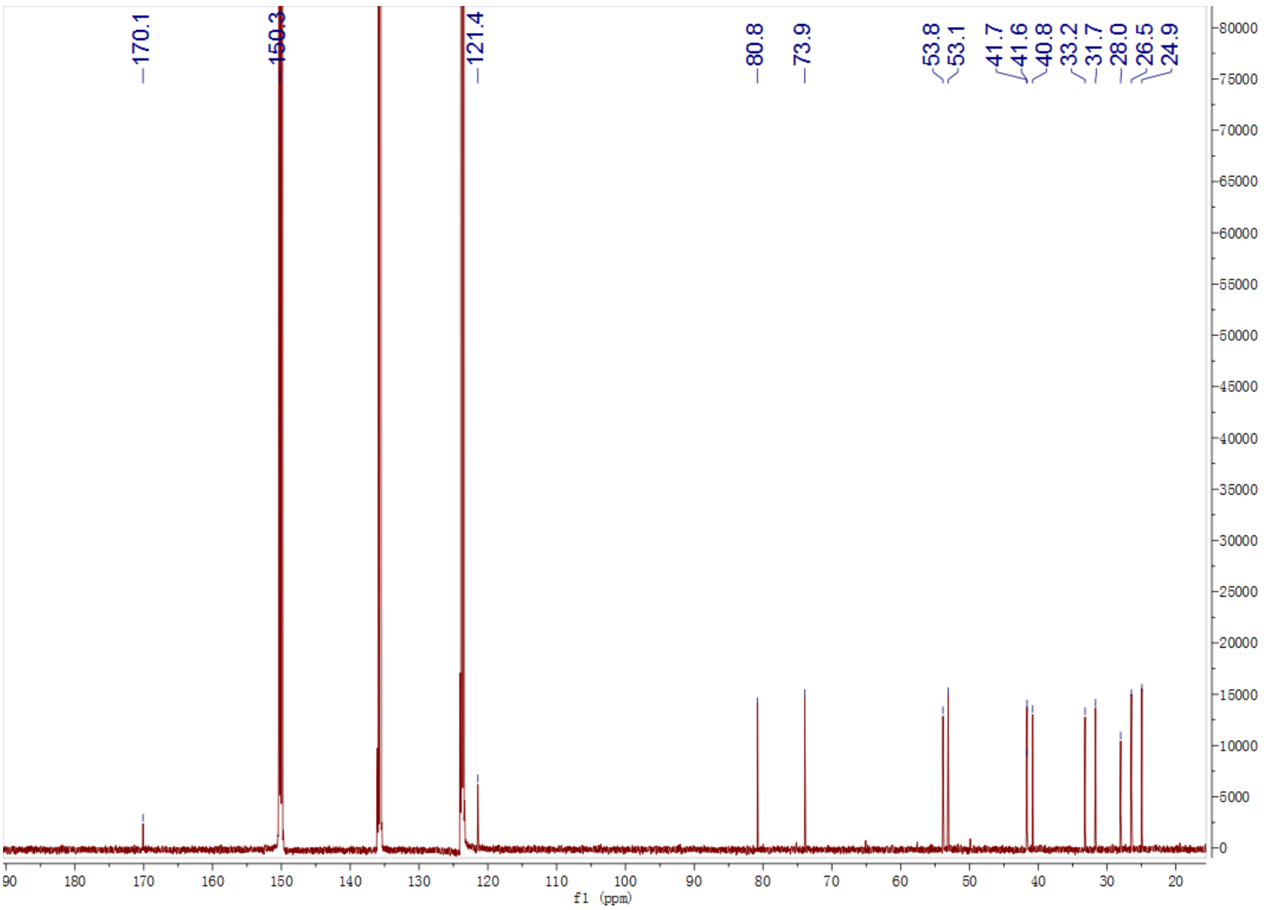

Figure S3. ^{13}C-NMR of compound **1**

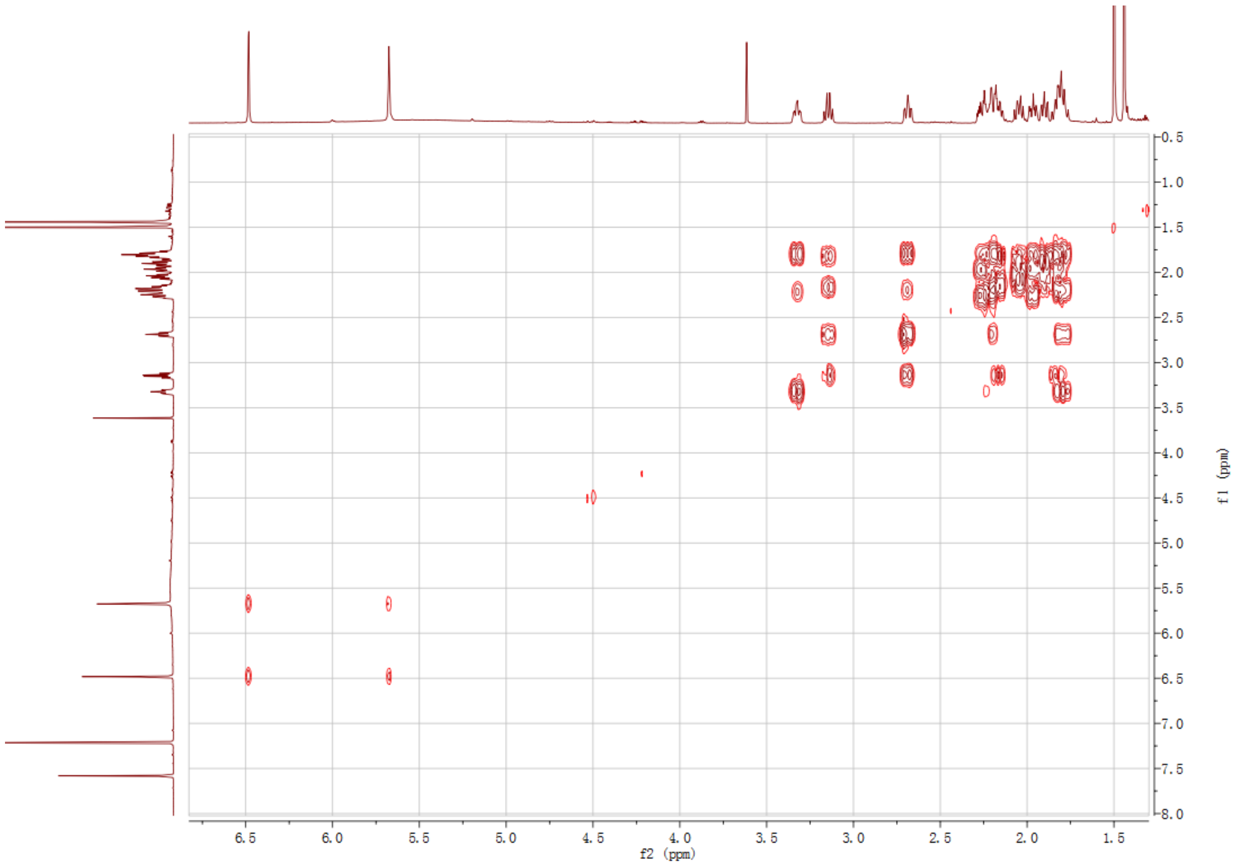

Figure S4. ^{1}H-^{1}H COSY of compound **1**

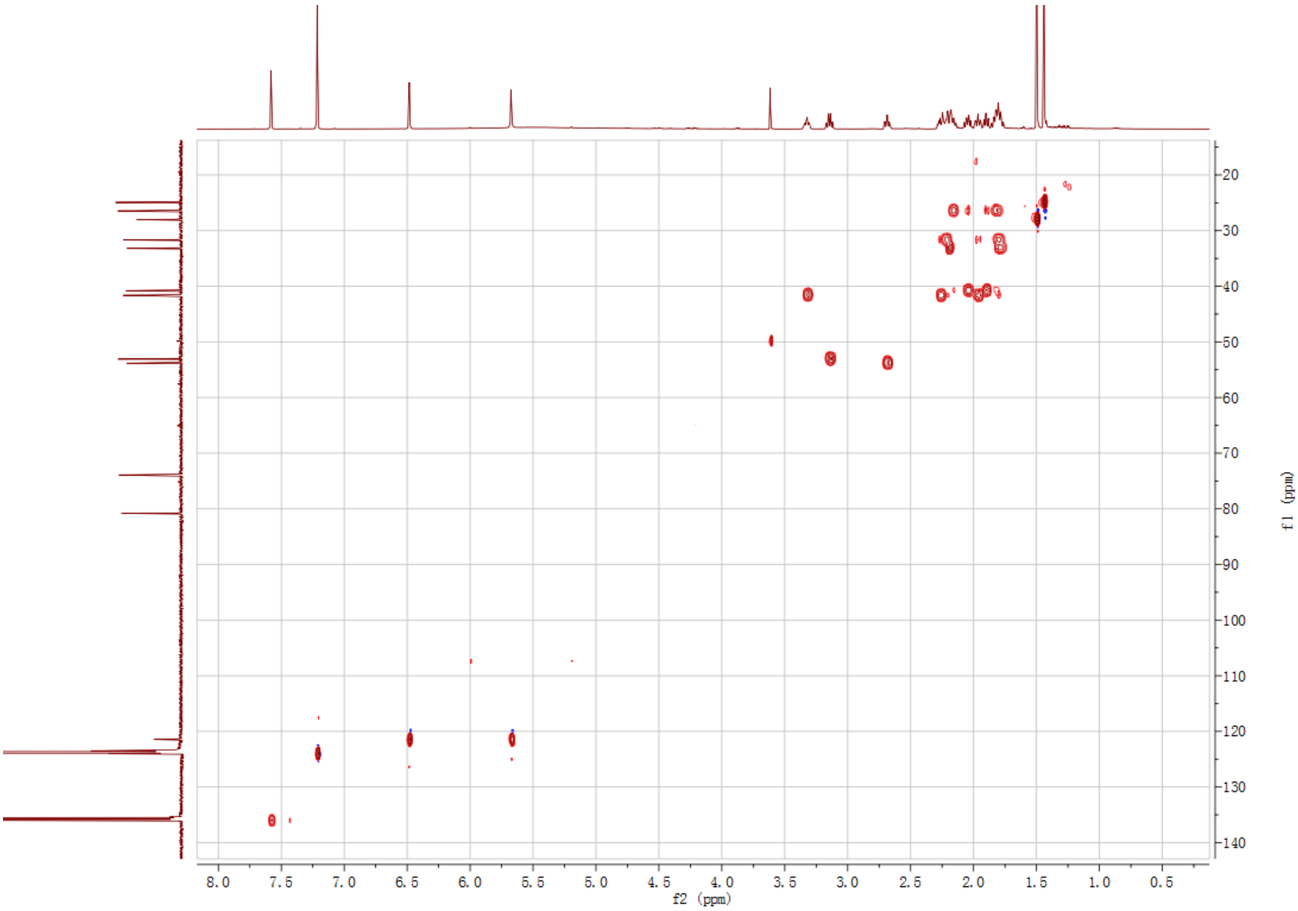

Figure S5. HSQC of compound **1**

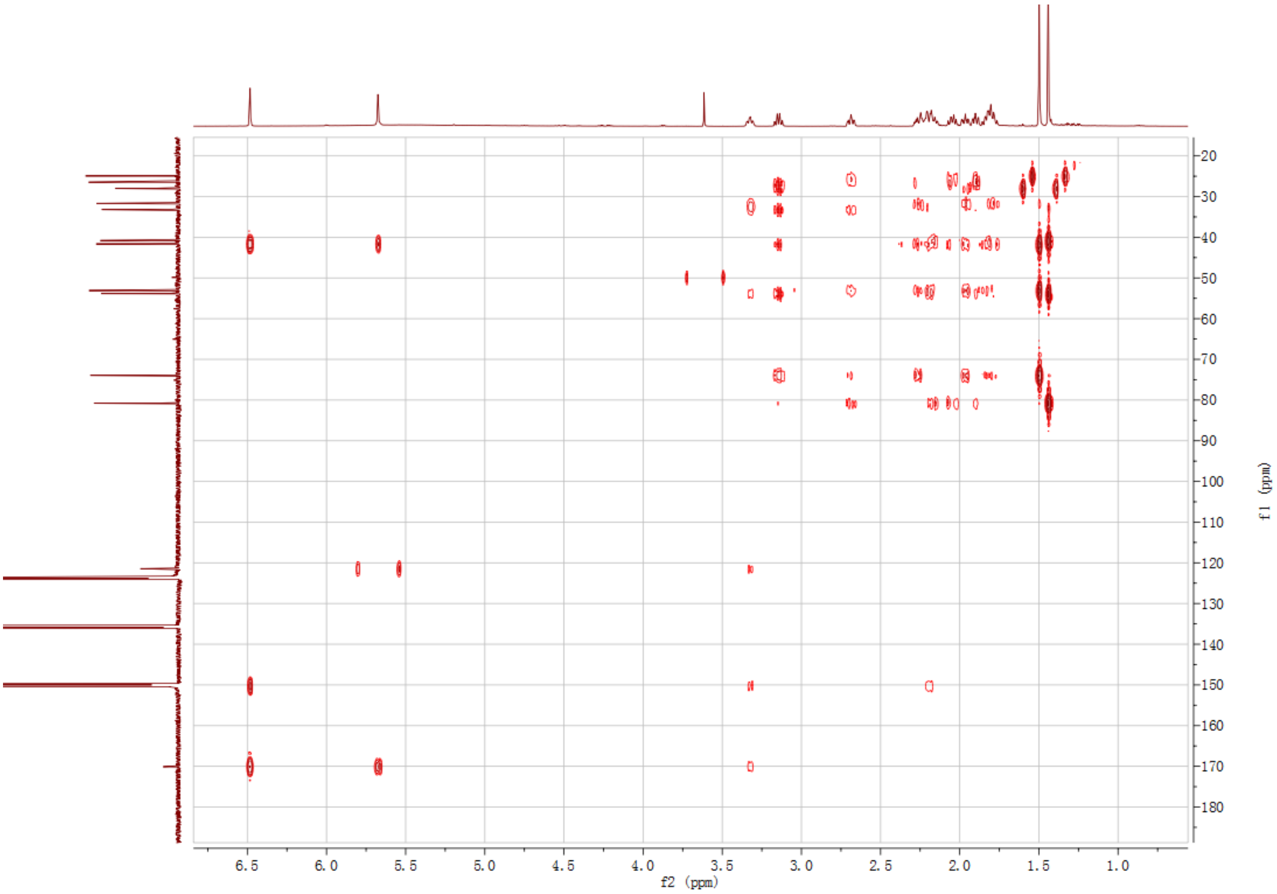

Figure S6. HMBC of compound **1**

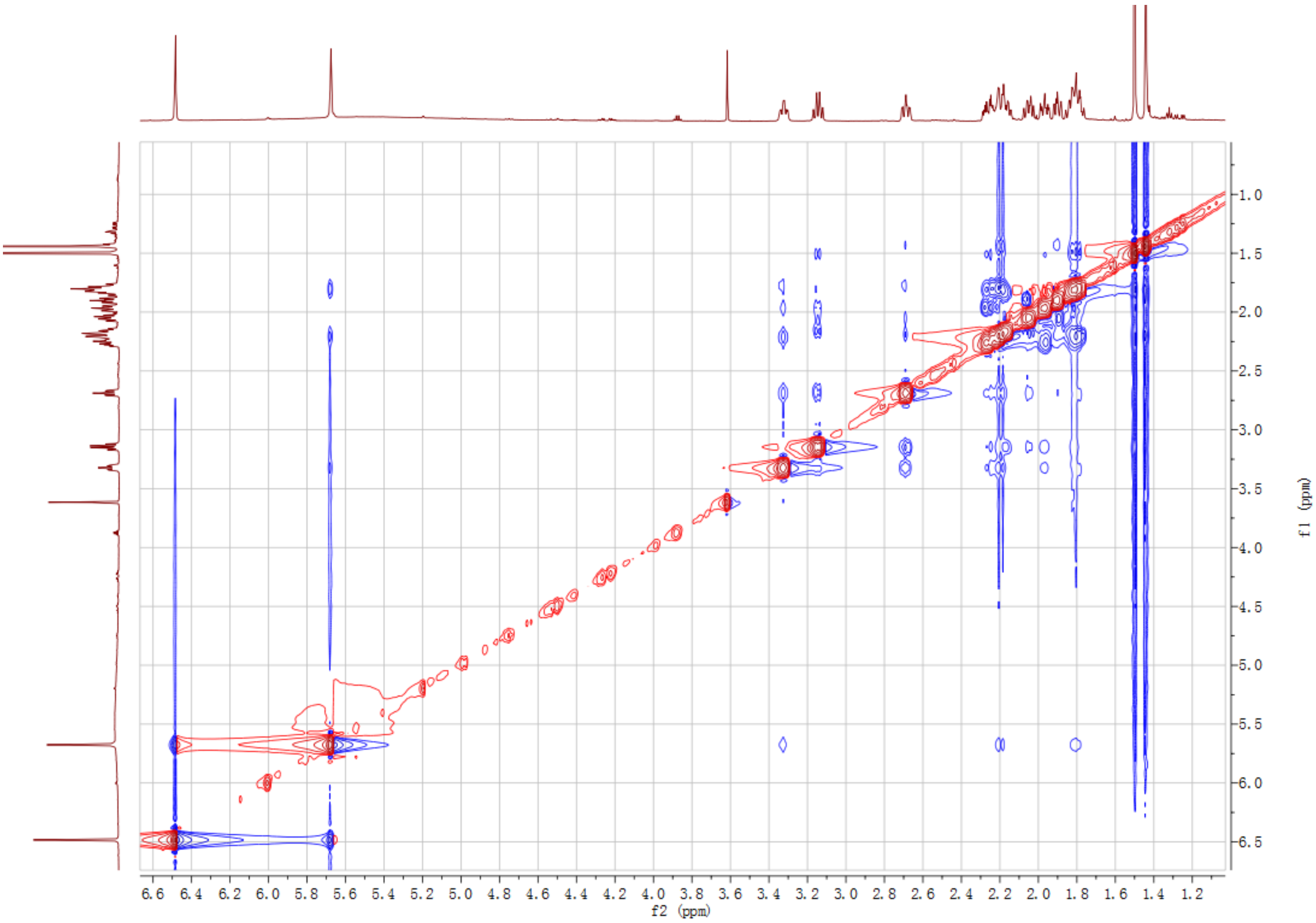

Figure S7. NOESY of compound **1**

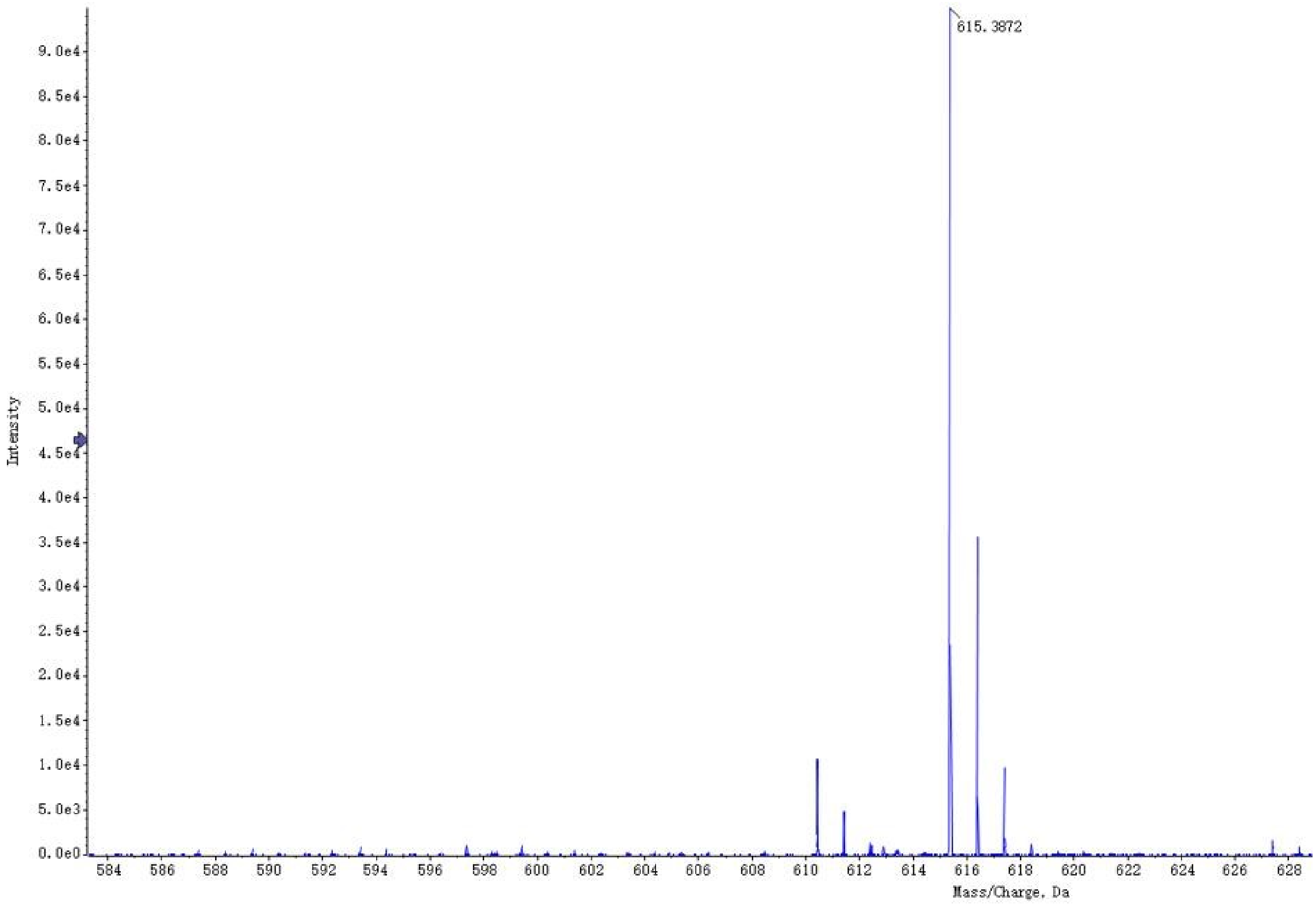

Figure S8. HR-ESIMS of compound **2**

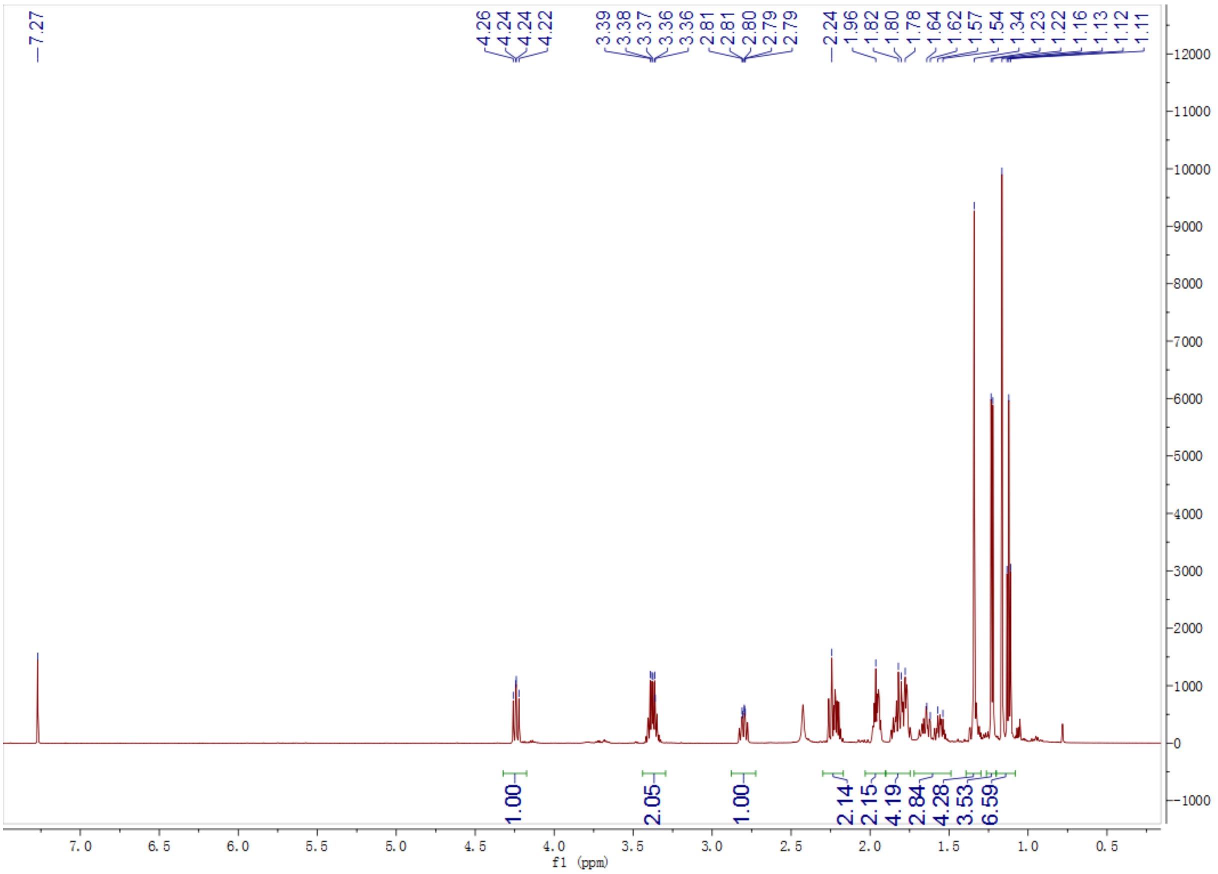

Figure S9. ^{1}H-NMR of compound **2**

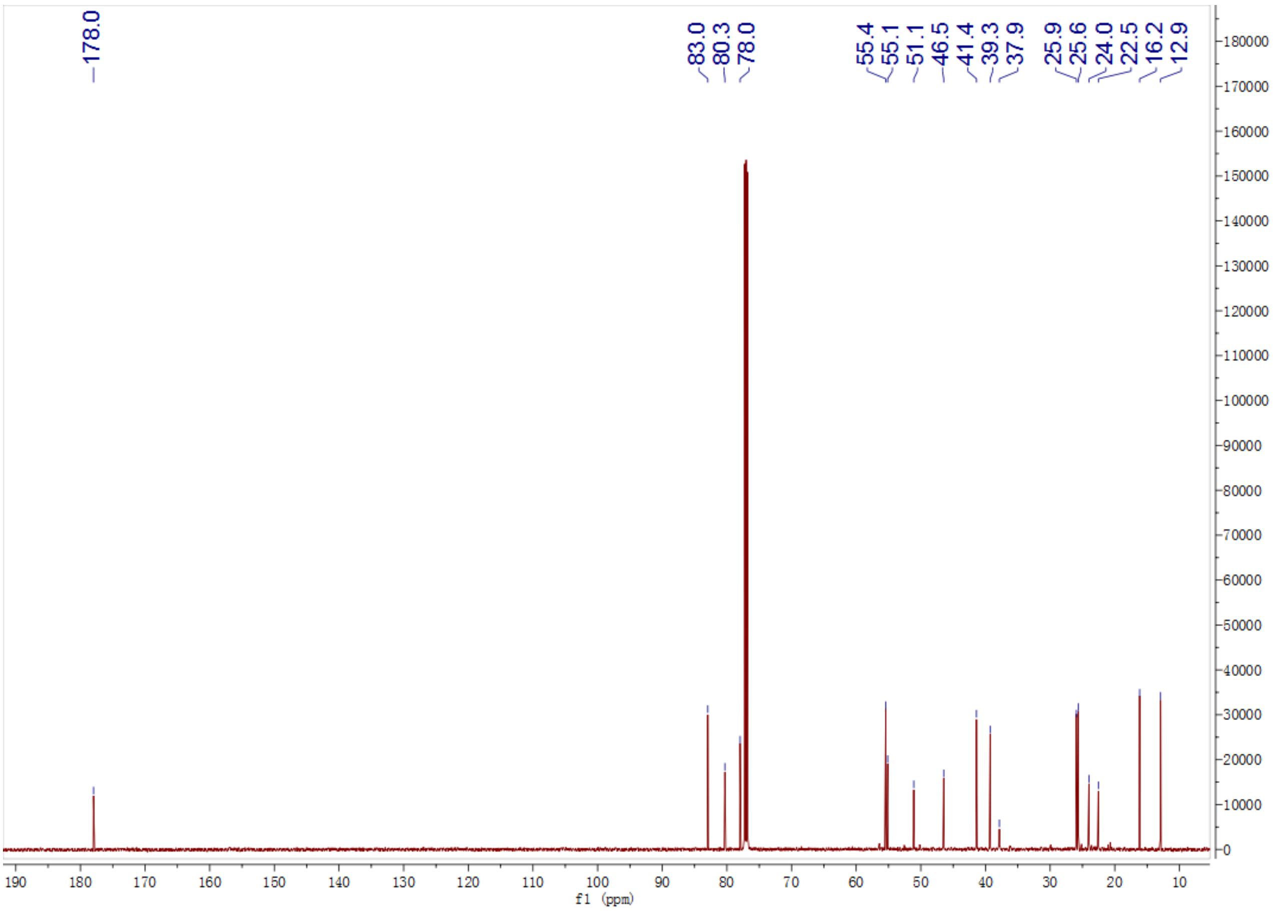

Figure S10. ^{13}C-NMR of compound **2**

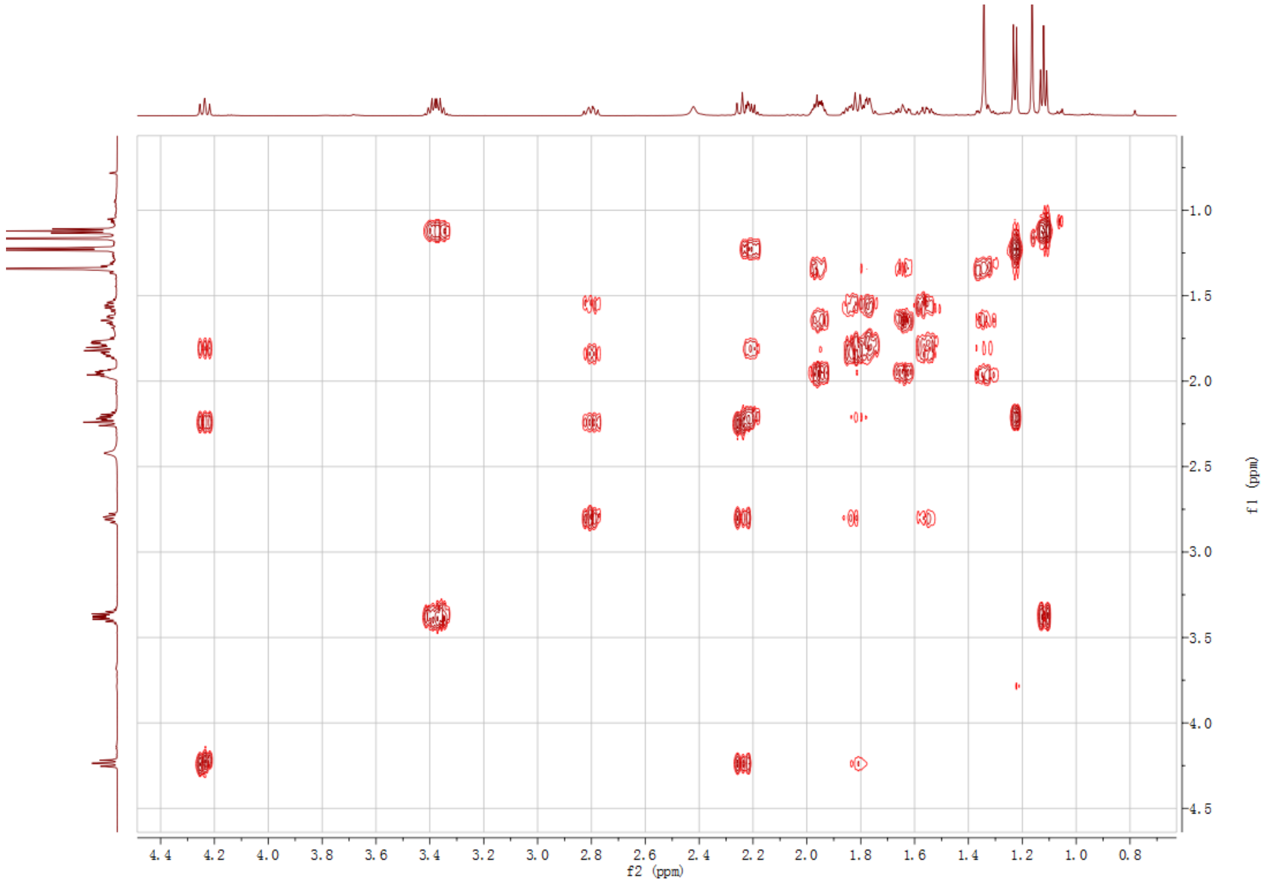

Figure S11. ^{1}H-^{1}H COSY of compound **2**

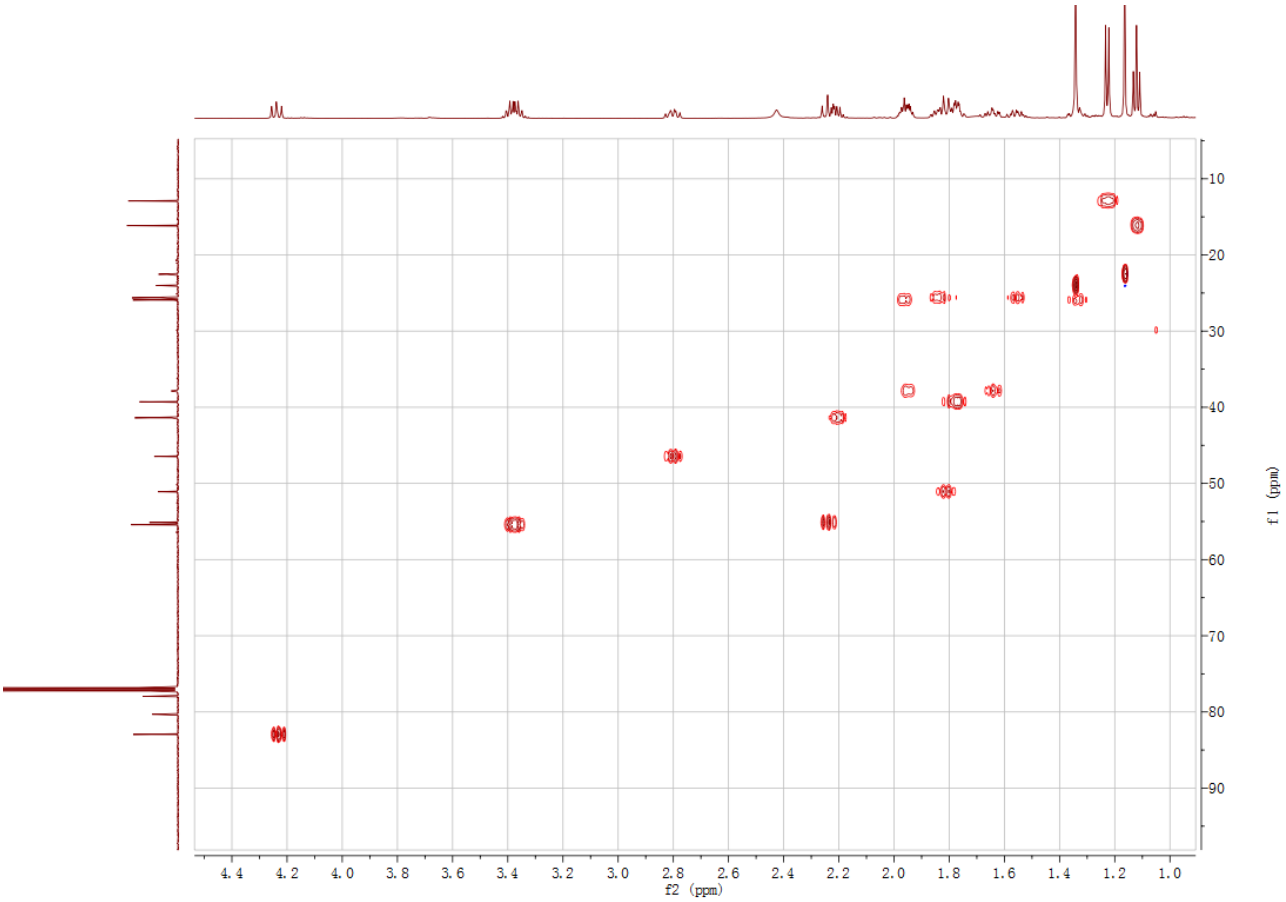

Figure S12. HSQC of compound **2**

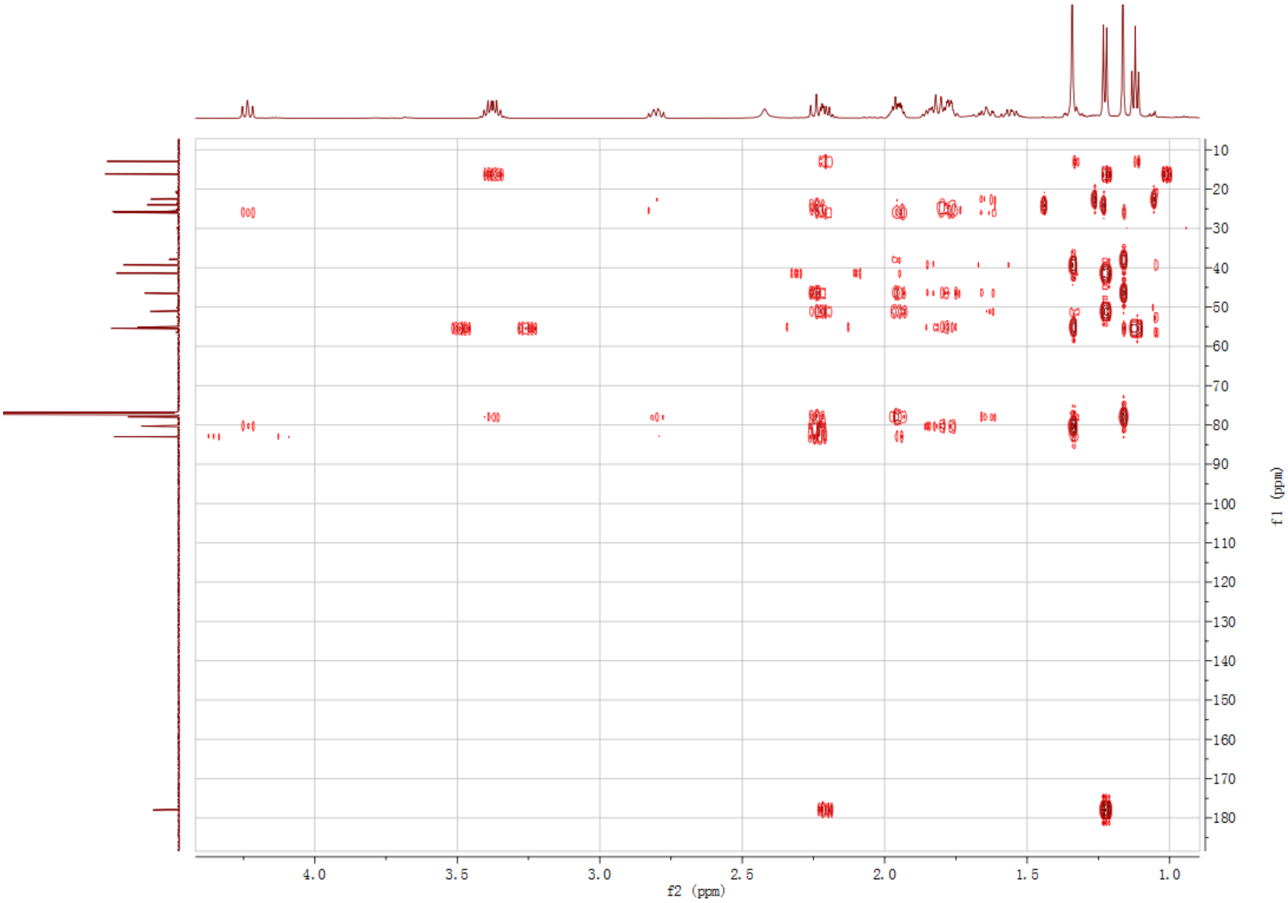

Figure S13. HMBC of compound **2**

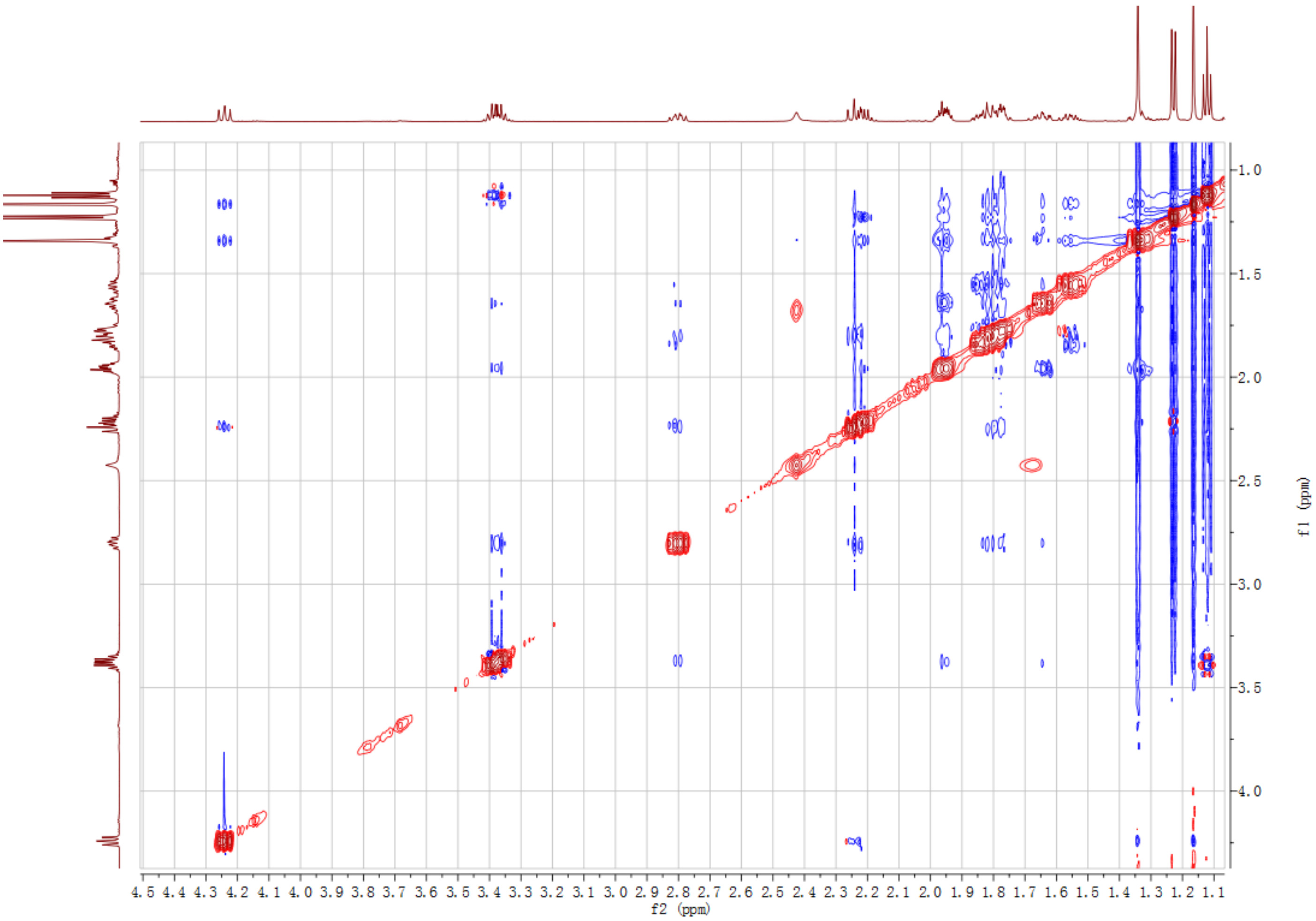

Figure S14. NOESY of compound **2**

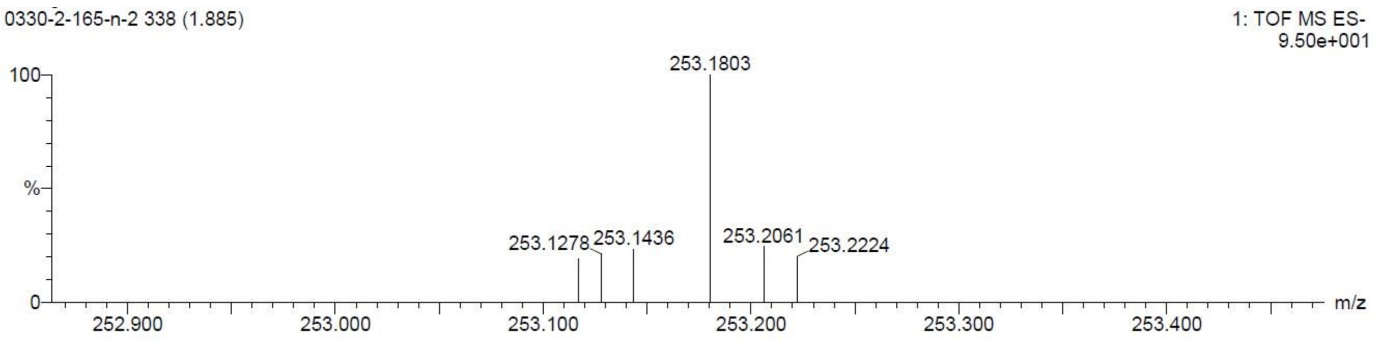

Figure S15. HR-ESIMS of compound **3**

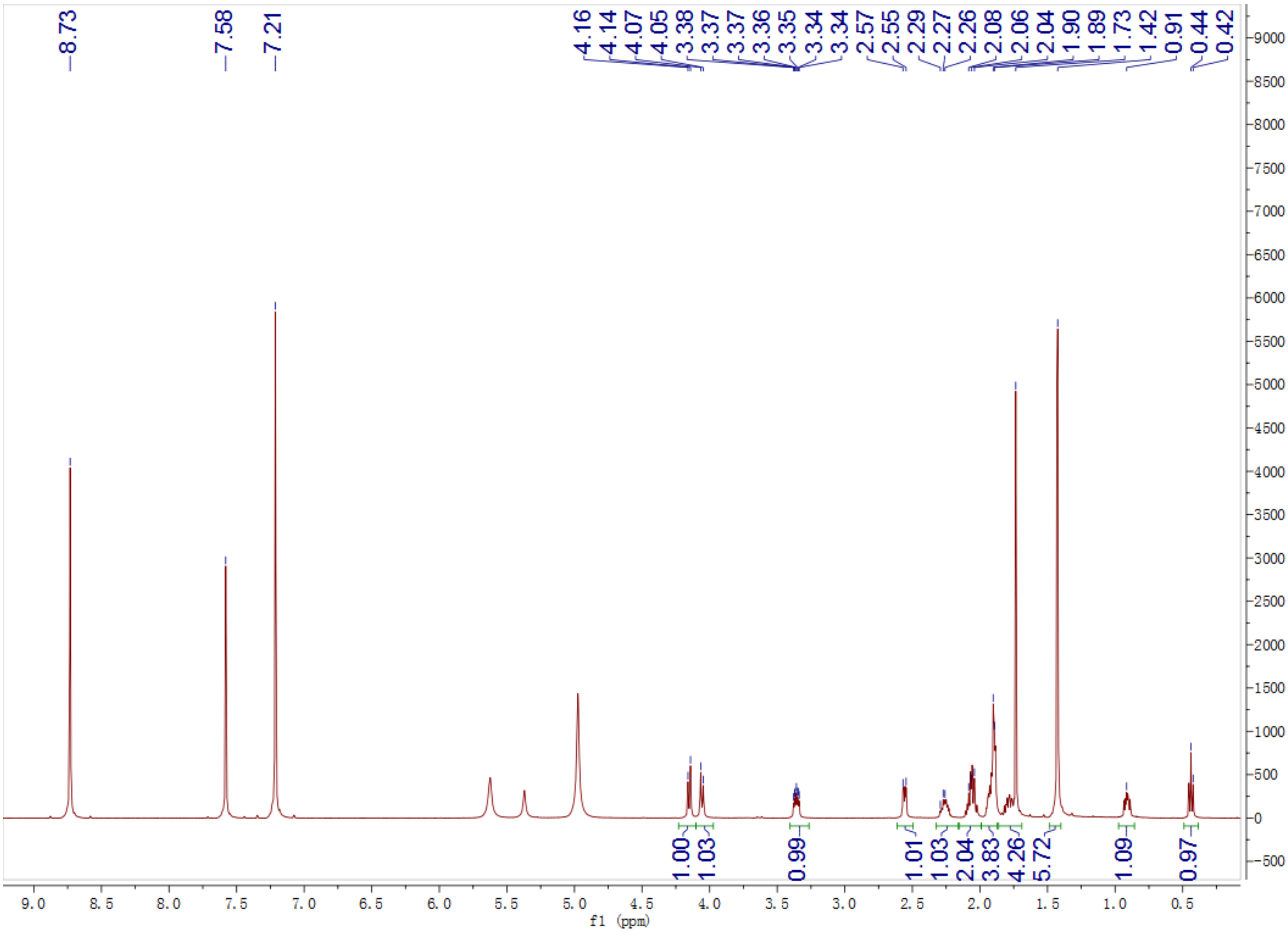

Figure S16. ^{1}H-NMR of compound **3**

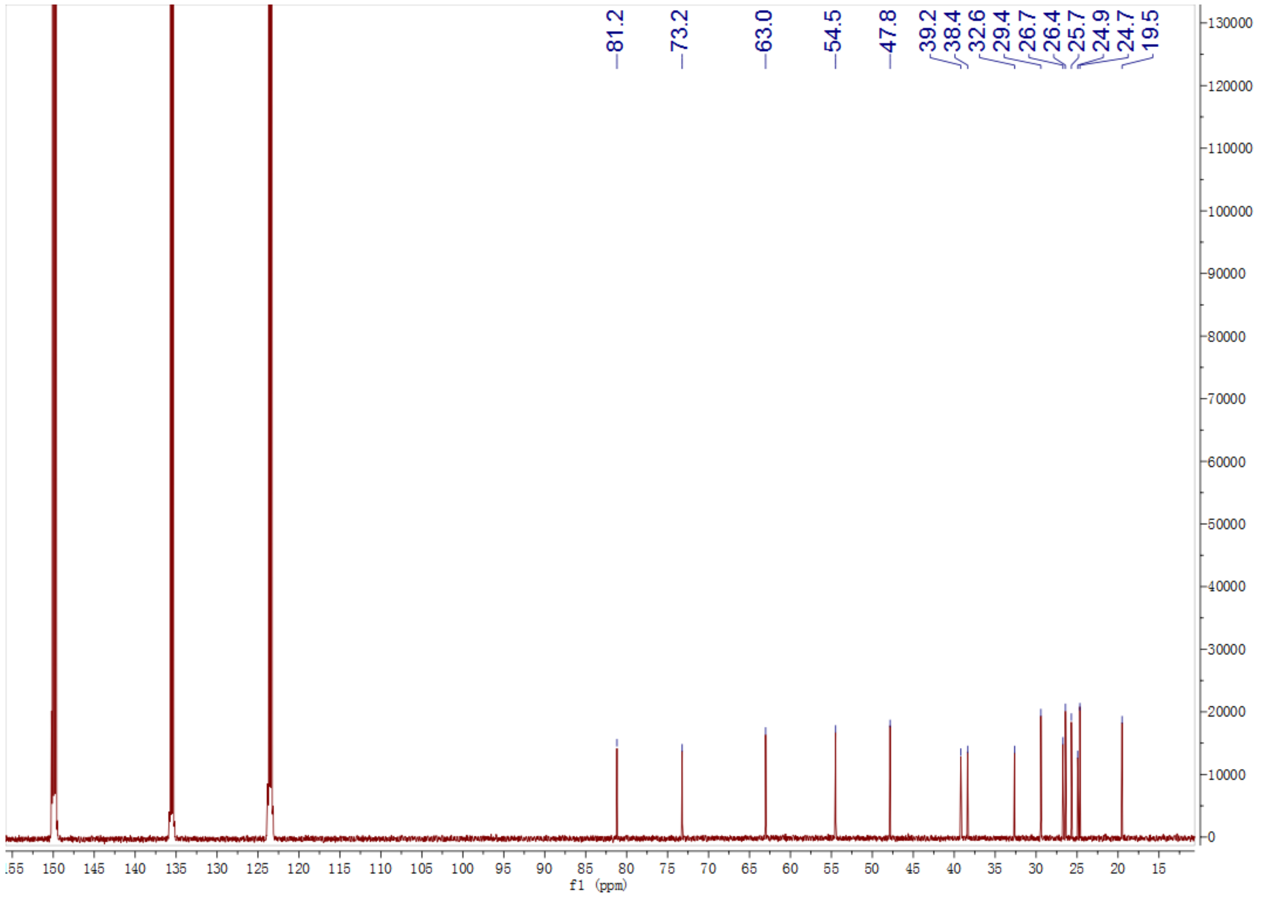

Figure S17. ^{13}C-NMR of compound **3**

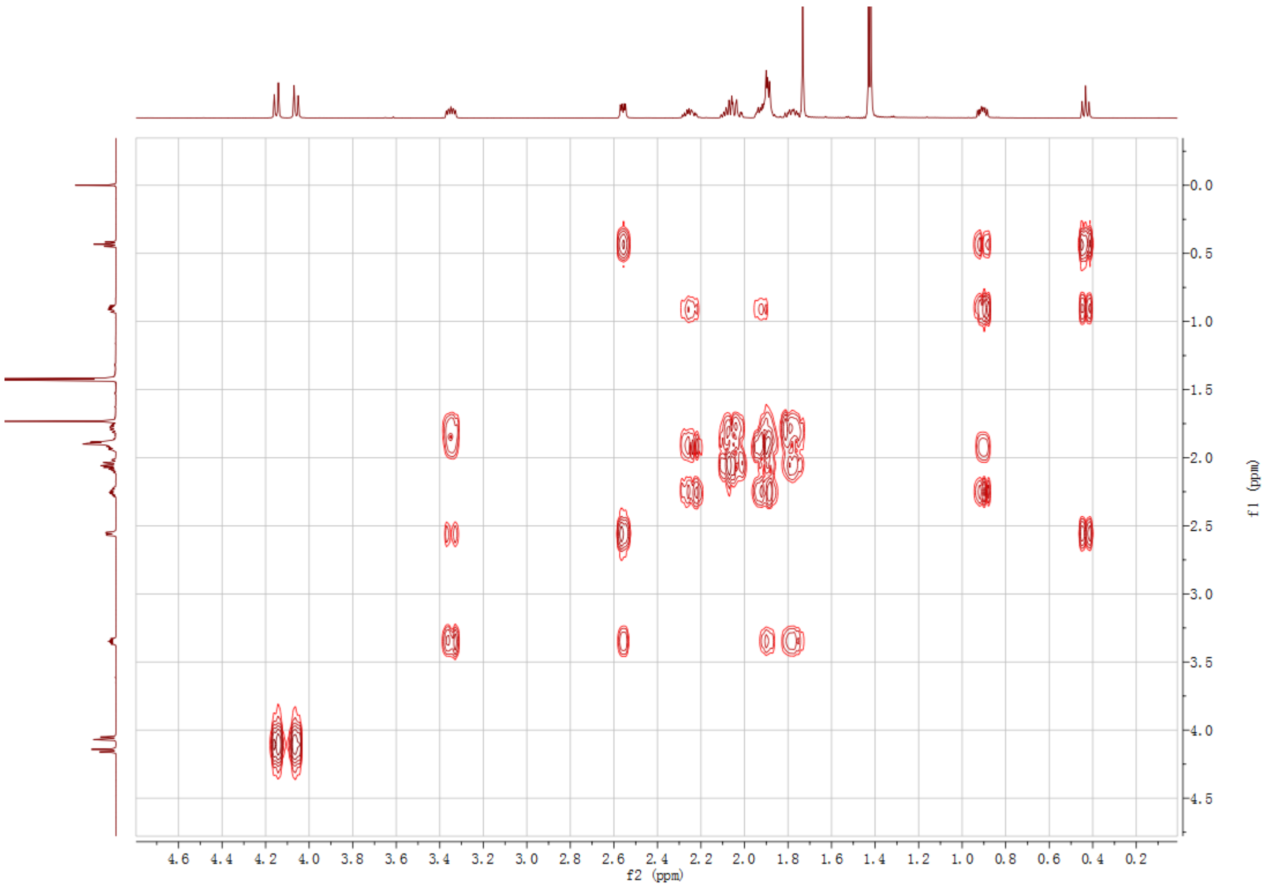

Figure S18. ^{1}H-^{1}H COSY of compound **3**

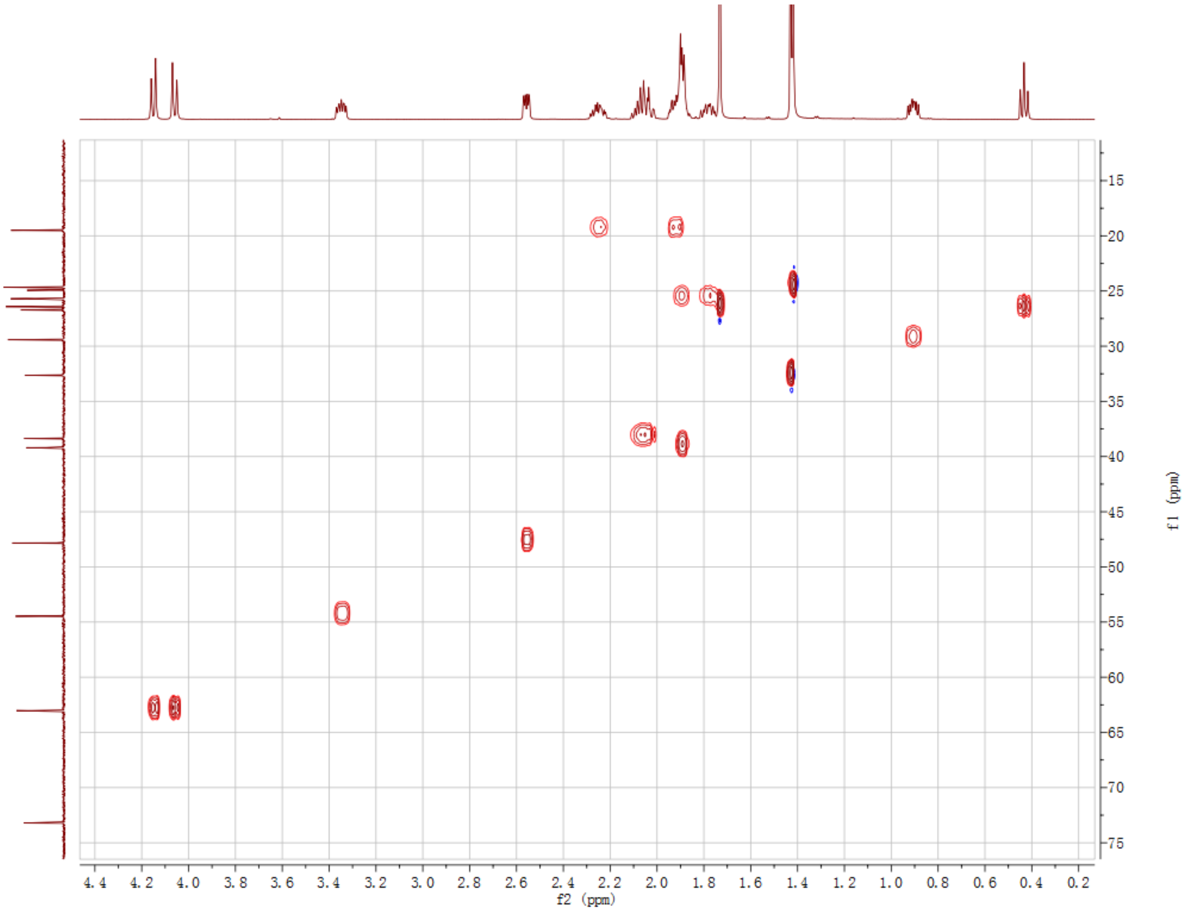

Figure S19. HSQC of compound **3**

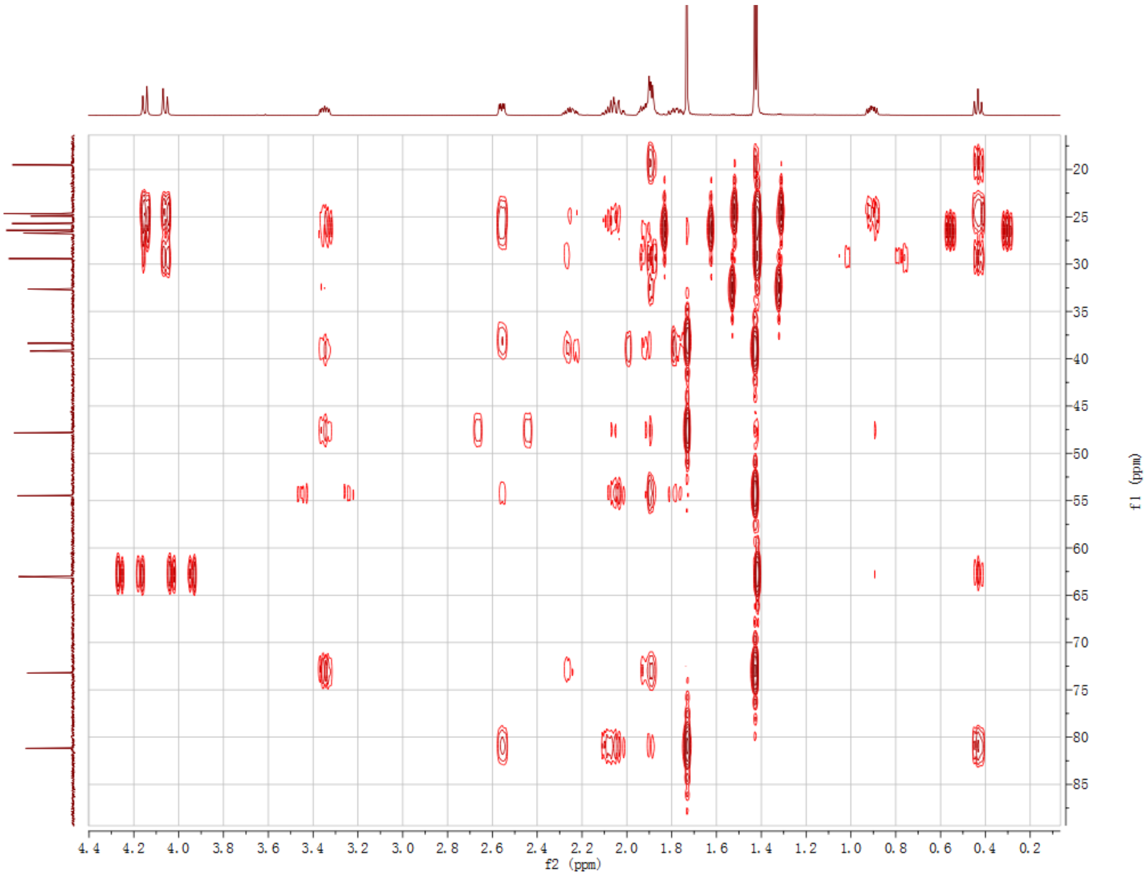

Figure S20. HMBC of compound **3**

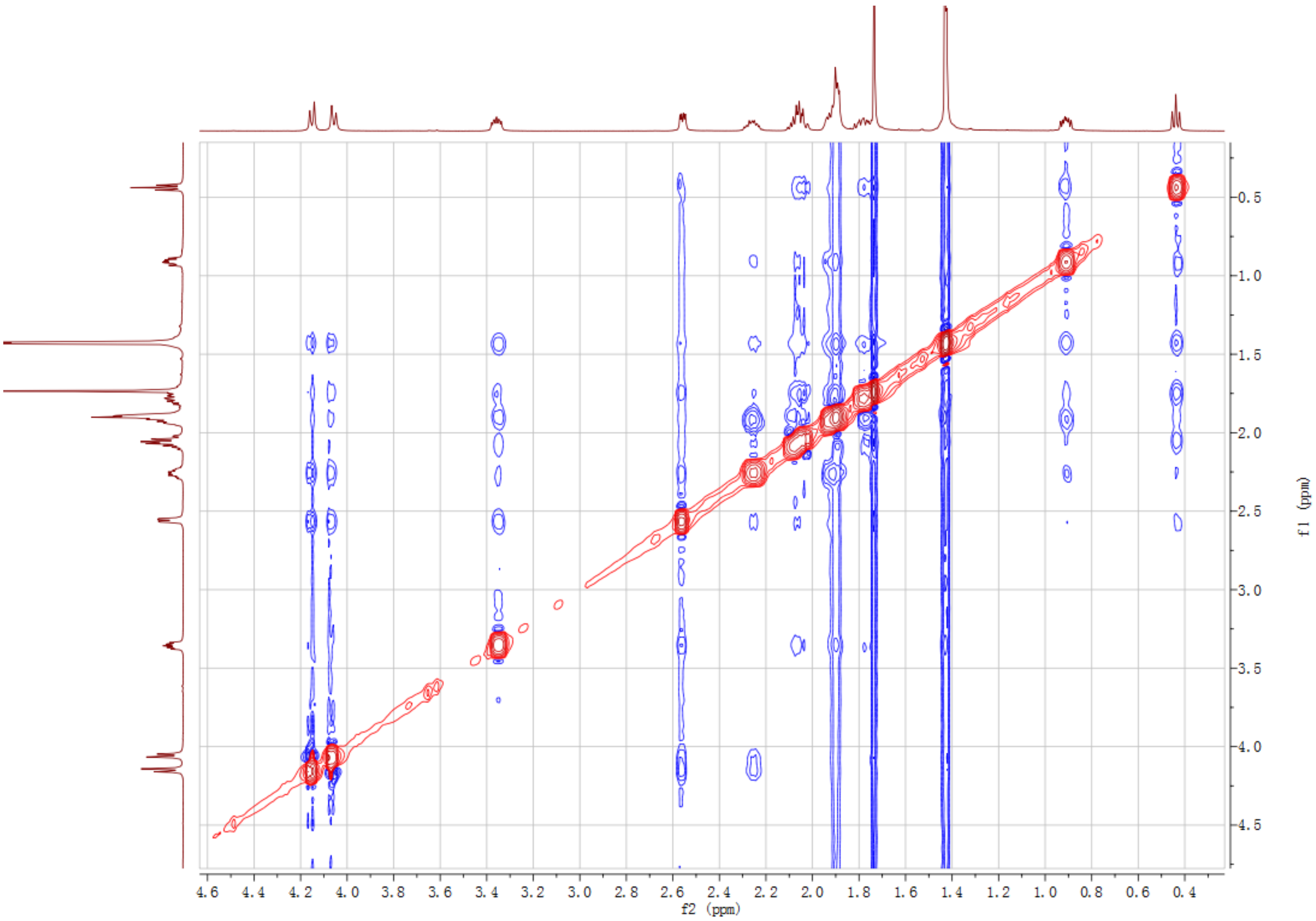

Figure S21. NOESY of compound **3**

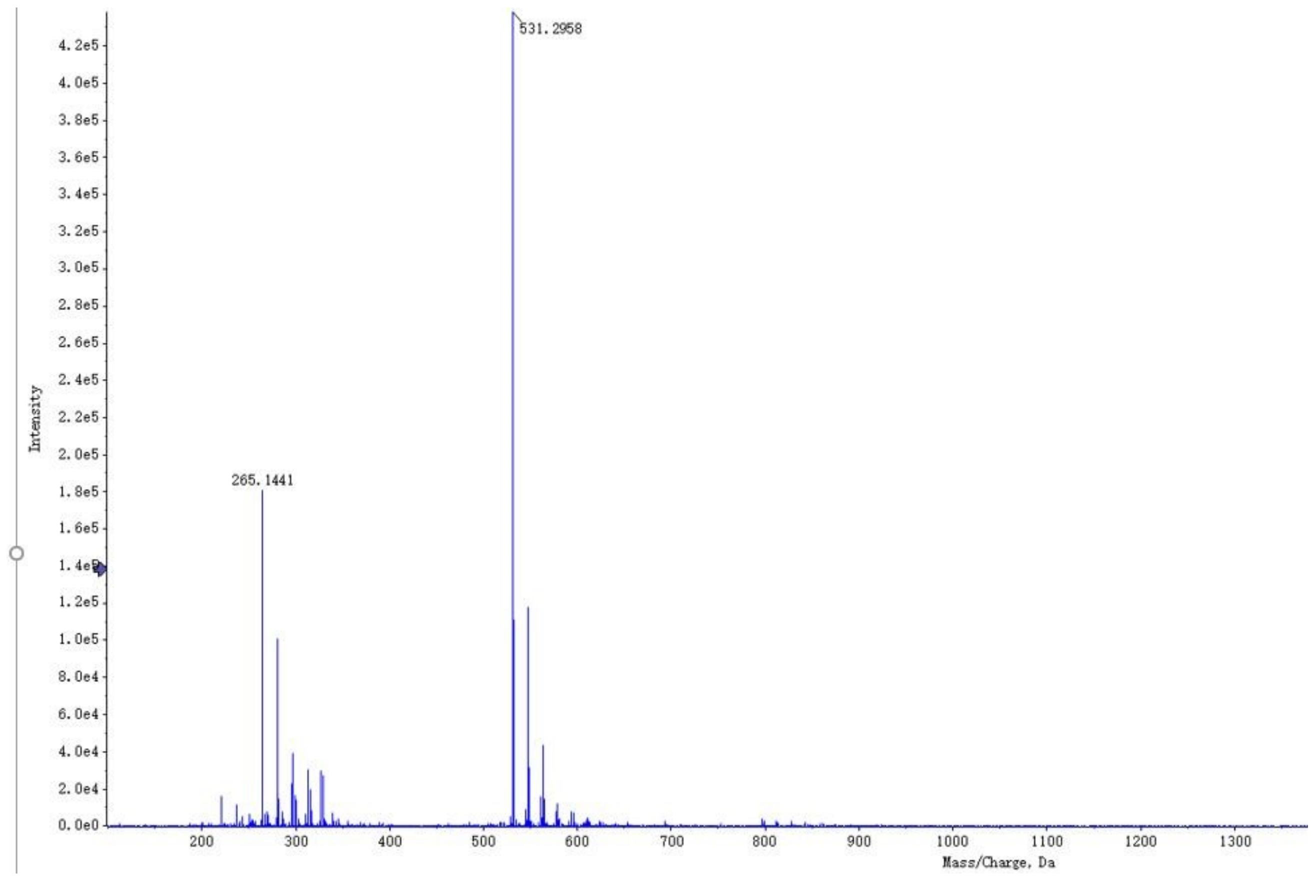

Figure S22. HR-ESIMS of compound **4**

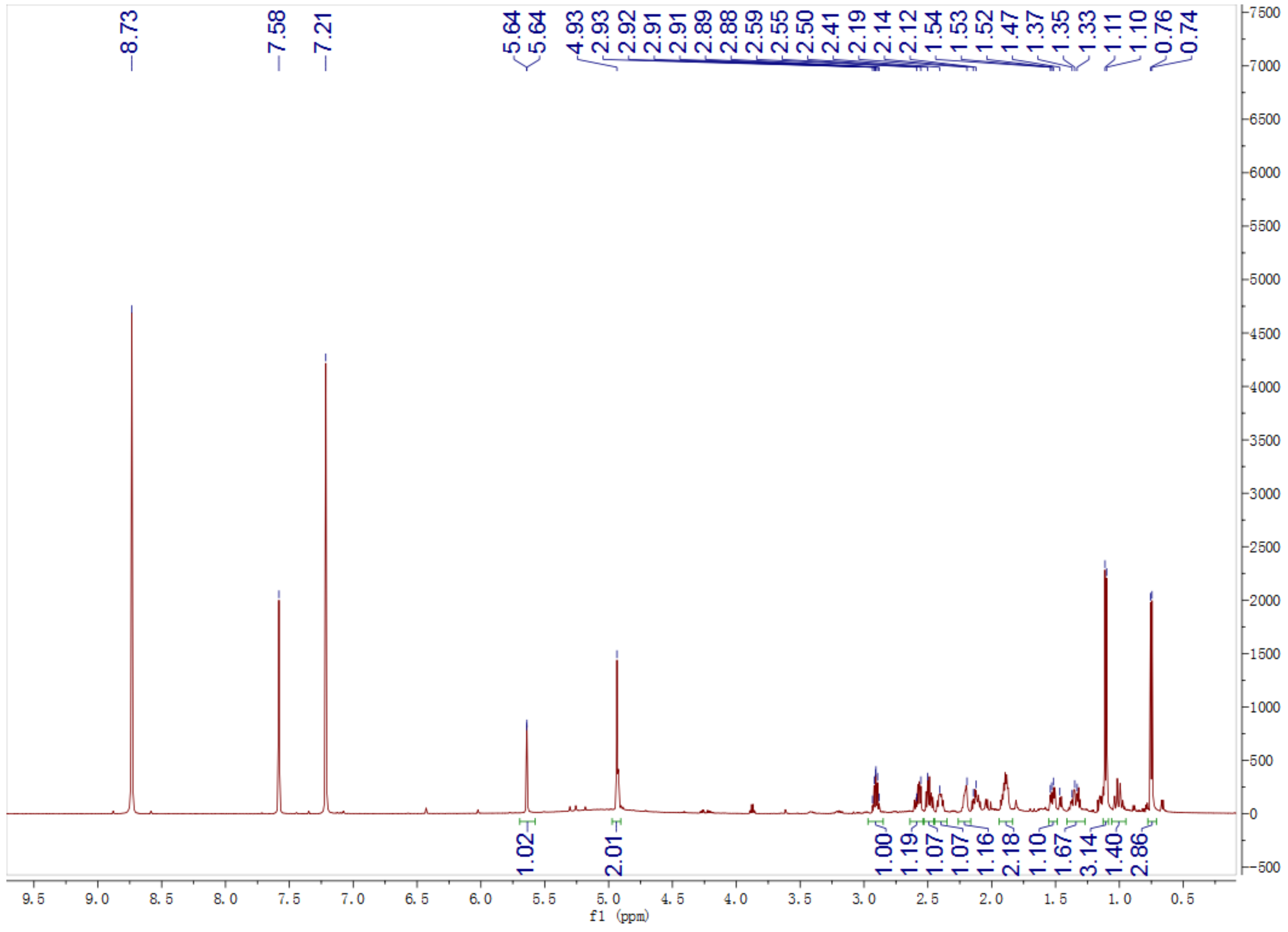

Figure S23. ^{1}H-NMR of compound **4**

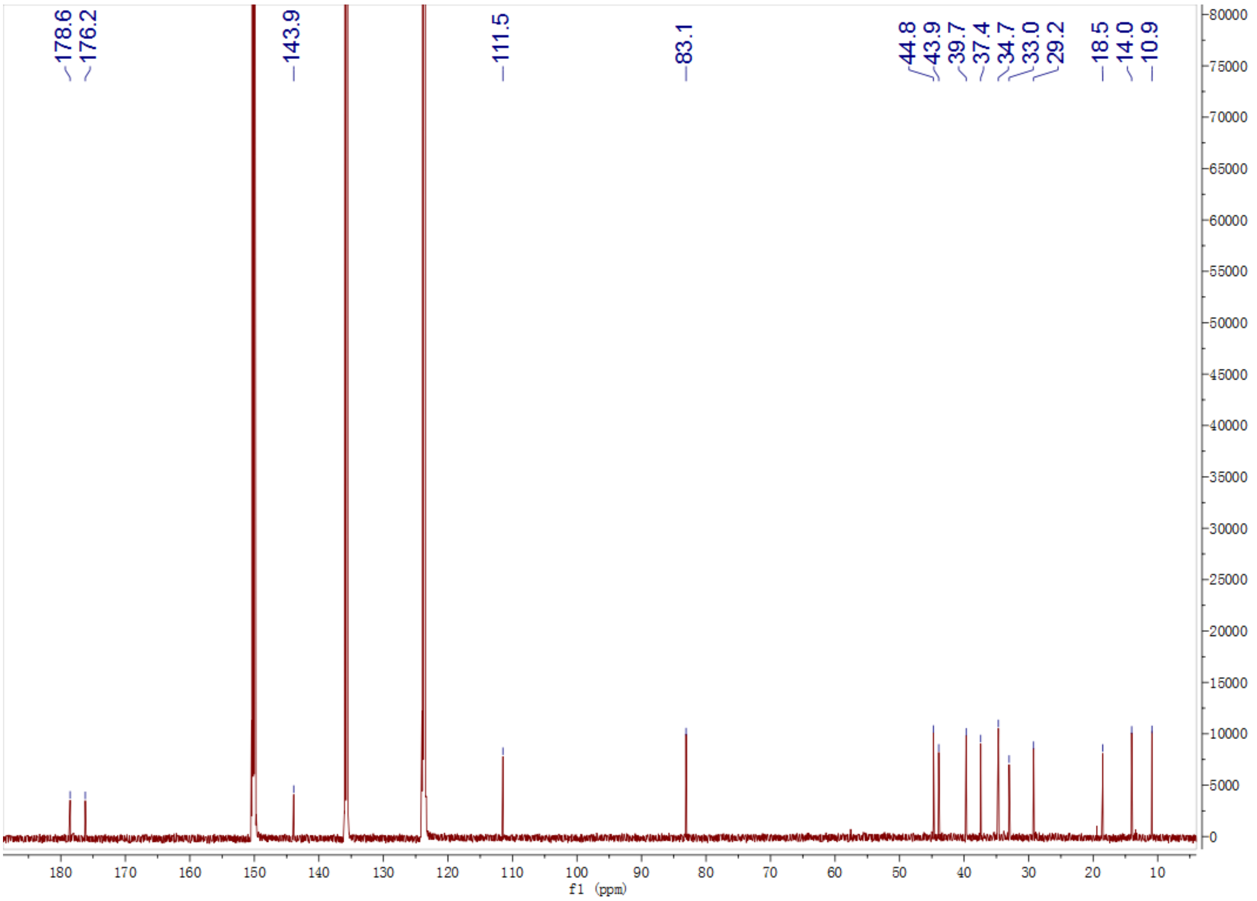

Figure S24. ^{13}C-NMR of compound **4**

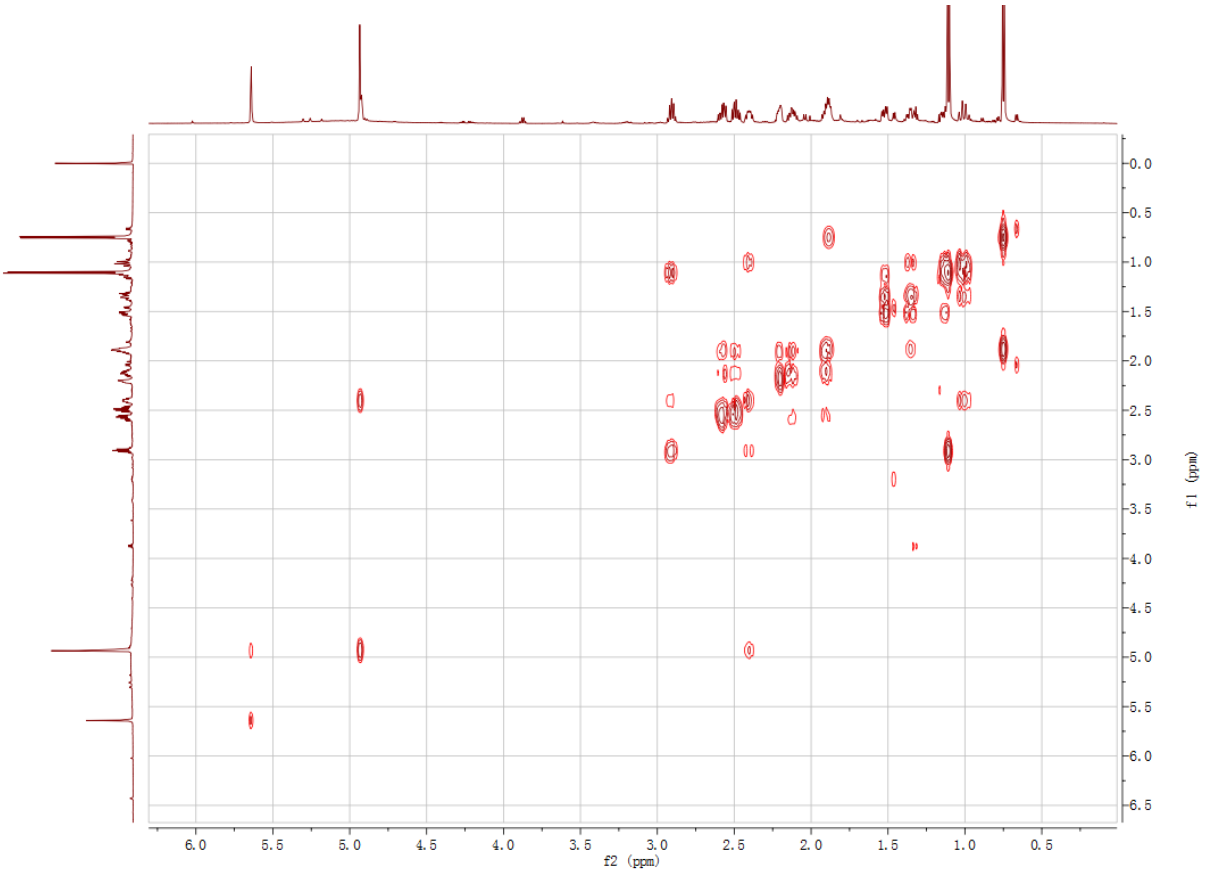

Figure S25. ^{1}H-^{1}H COSY of compound **4**

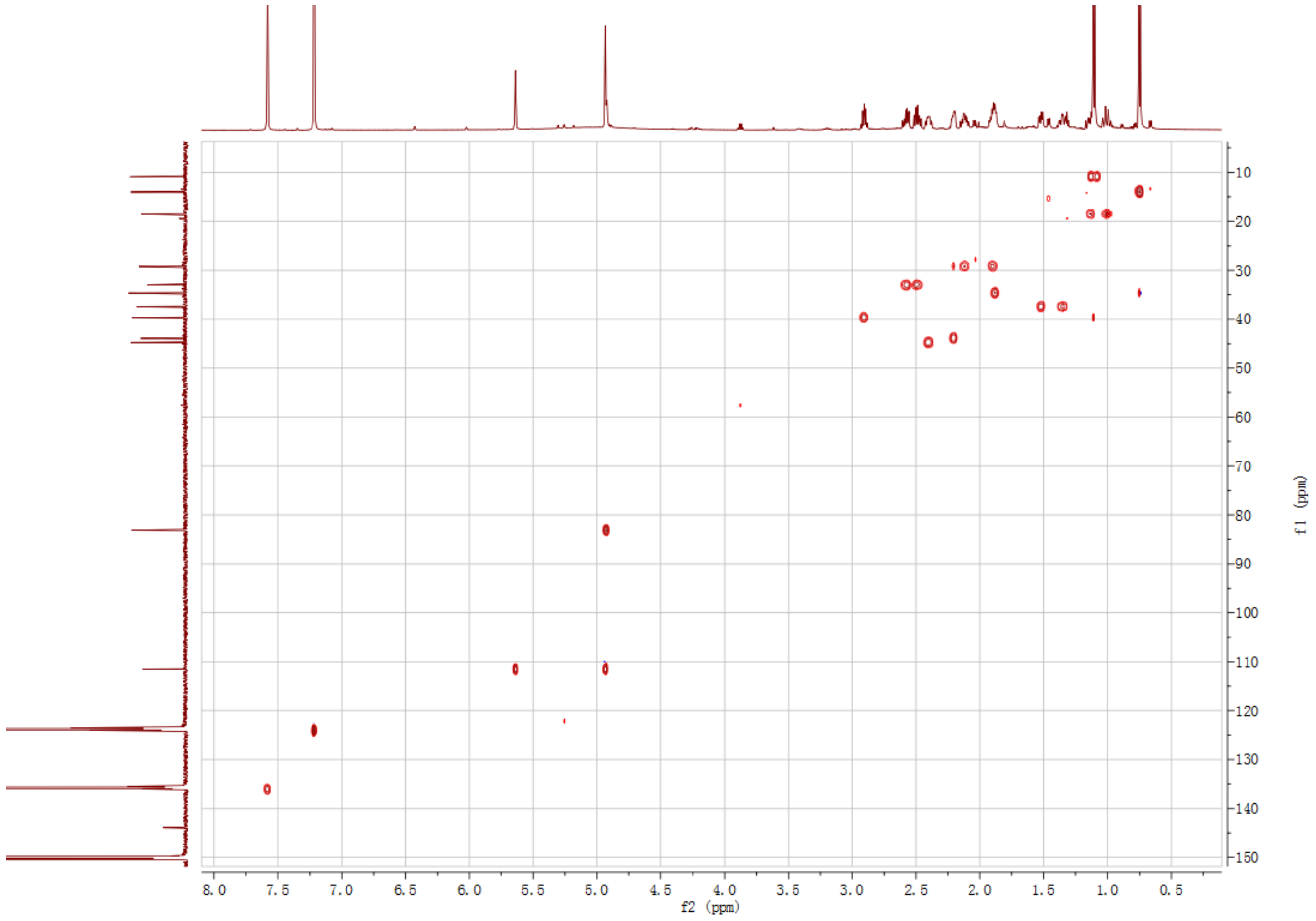

Figure S26. HSQC of compound **4**

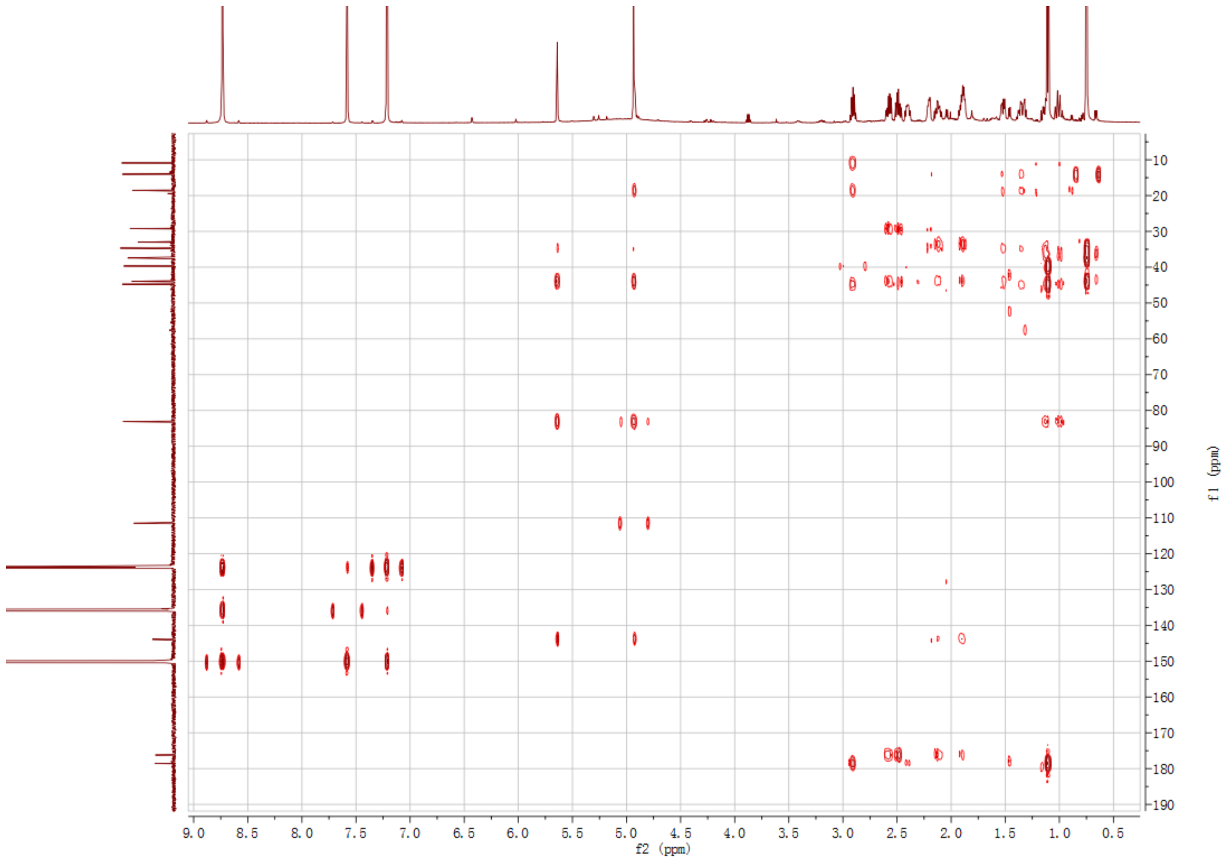

Figure S27. HMBC of compound **4**

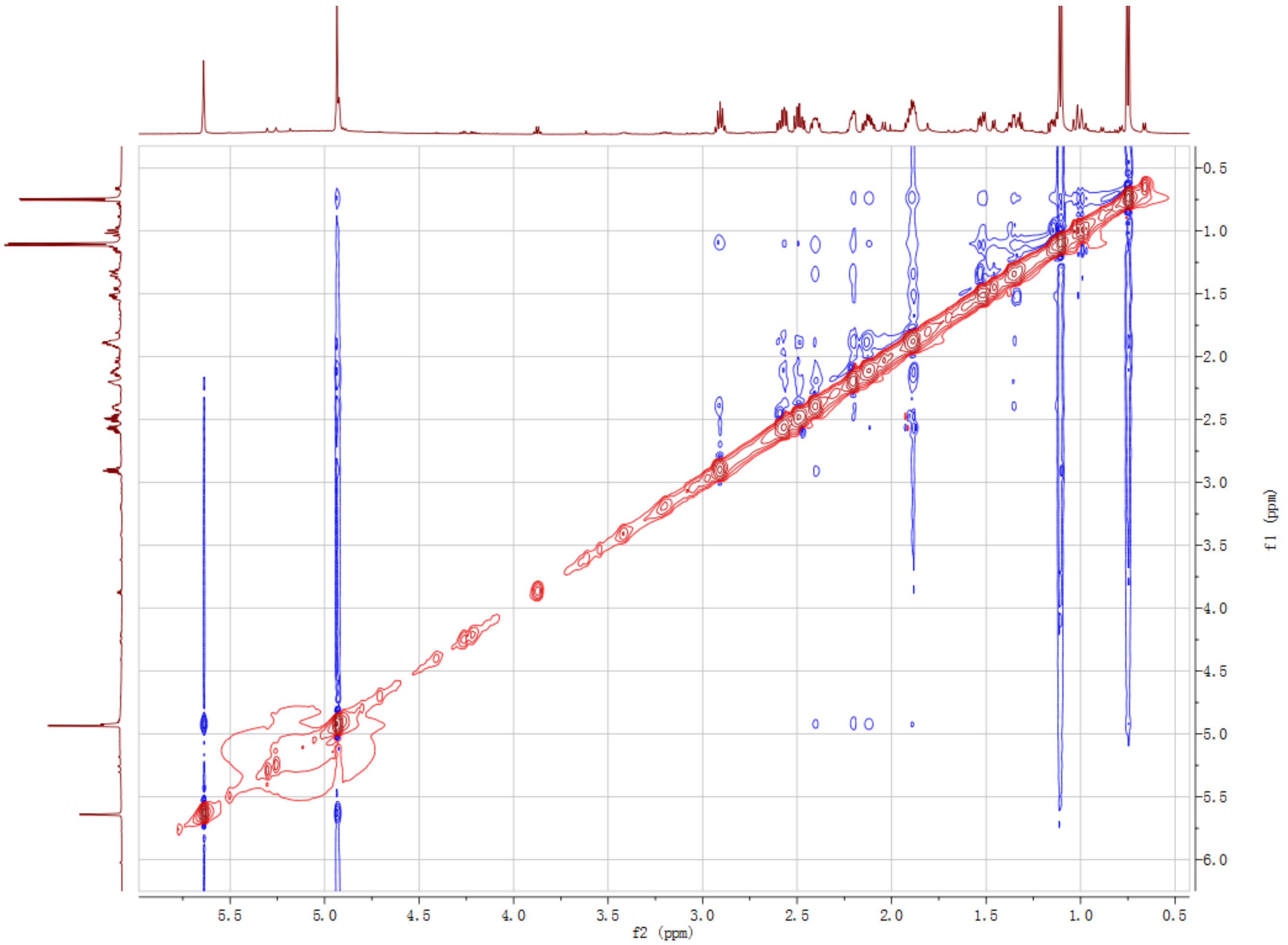

Figure S28. NOESY of compound **4**
